# Bioresponsive gelatin-coated silk fibroin nanoparticles enhance roxadustat therapy in experimental pulmonary fibrosis

**DOI:** 10.3389/fphar.2026.1921860

**Published:** 2026-08-03

**Authors:** Elsayed A. Elmorsy, Sameh Saber, Abdulaziz A. Alsalloom, Abdullah Al-Nafeesah, Mostafa M. Khodeir, Abdullah Alolayan, Adel Alhomaidi, Mohammed Alorini, Mohamed R. Abdel-Hamed, Maha M. Amer, Huda Eltayeb, Syed Suhail Ahmed, Noura El Adle Khalaf, Eman Hamza, Ebtehal M. Abdel-Aal, Abdelrahman El-Sayed, Ahmed Y. Kira

**Affiliations:** 1 Department of Pharmacology and Toxicology, College of Pharmacy, Qassim University, Buraidah, Saudi Arabia; 2 Department of Clinical Pharmacology, Faculty of Medicine, Mansoura University, Mansoura, Egypt; 3 Department of Pharmacology, Faculty of Pharmacy, Delta University for Science and Technology, Mansoura, Egypt; 4 Department of Pathology, College of Medicine, Qassim University, Buraidah, Saudi Arabia; 5 Department of Pediatrics, College of Medicine, Qassim University, Buraidah, Saudi Arabia; 6 Department of Pathology, Faculty of Medicine, Cairo University, Cairo, Egypt; 7 Department of Anatomy and Histology, College of Medicine, Qassim University, Buraidah, Saudi Arabia; 8 Department of Anatomy and Embryology, Faculty of Medicine, Ain Shams University, Cairo, Egypt; 9 Department of Basic Sciences, College of Medicine, Princess Nourah bint Abdulrahman University, Riyadh, Saudi Arabia; 10 Department of Microbiology and Immunology, College of Medicine, Qassim University, Buraidah, Saudi Arabia; 11 Department of Biochemistry, College of Medicine, Imam Mohammad Ibn Saud Islamic University (IMSIU), Riyadh, Saudi Arabia; 12 Department of Pathology, College of Medicine, Imam Mohammad Ibn Saud Islamic University (IMSIU), Riyadh, Saudi Arabia; 13 Faculty of Medicine, Damietta University, New Damietta, Egypt; 14 Department of Pharmaceutics, Faculty of Pharmacy, Delta University for Science and Technology, Mansoura, Egypt

**Keywords:** gelatin coating, MMP-2-responsive delivery, pulmonary fibrosis, roxadustat, silk fibroin nanoparticles

## Abstract

Pulmonary fibrosis (PF) requires delivery strategies that improve drug residence within lung tissue while limiting systemic exposure. Herein, roxadustat-loaded silk fibroin nanoparticles (RXS-SKNPs) were developed and coated with gelatin (GT) to produce an MMP-2-responsive pulmonary delivery platform. RXS-SKNPs were prepared by desolvation-assisted nanoprecipitation, followed by GT coating via electrostatic interactions. The optimized GT-RXS-SKNPs showed an increase in particle size from 145.72 ± 2.74 nm to 194.26 ± 2.94 nm, a zeta-potential shift from −30.28 ± 1.01 mV to −15.19 ± 2.38 mV, and an entrapment efficiency of 72.11% ± 0.81%. FTIR analysis and the increased surface-accessible amino-group signal confirmed GT coating and RXS incorporation. GT-RXS-SKNPs exhibited MMP-2-triggered colloidal remodeling and enzyme-responsive release, with cumulative RXS release increasing to 59.93% ± 1.30% after 24 h under MMP-2 exposure compared with 43.70% ± 1.32% in enzyme-free medium. Release followed anomalous non-Fickian behavior, indicating combined diffusion and GT shell erosion/relaxation. *In vivo*, GT-RXS-SKNPs reduced plasma exposure while increasing lung AUC_0_–_24_ by 3.58-fold and 1.71-fold compared with free RXS and uncoated RXS-SKNPs, respectively, and prolonged lung MRT to 42.13 ± 8.34 h. In bleomycin-induced PF, GT-RXS-SKNPs produced the strongest attenuation of histopathological injury, collagen deposition, myofibroblast activation, BALF inflammatory cytokines, lung hydroxyproline, TGF-β1, MMP-2, MMP-9, lipid peroxidation, and antioxidant depletion. Integrated recovery analysis showed the highest overall fibrosis recovery index for GT-RXS-SKNPs (85.3%), outperforming uncoated RXS-SKNPs (60.8%) and free RXS (34.1%). Overall, GT-RXS-SKNPs improved pulmonary retention and translated enhanced lung delivery into superior antifibrotic efficacy while limiting systemic exposure.

## 1 Introduction

Pulmonary fibrosis (PF) is a progressive and fatal interstitial lung disease. PF is characterized by persistent alveolar damage, excessive deposition of extracellular matrix (ECM), collagen accumulation, architectural distortion, and progressive impairment of gas exchange (Piera-Velazquez et al., 2011; Deng et al., 2020). Over time, the fibrotic process reduces lung compliance and may lead to respiratory failure (McDonald, 2021). While pirfenidone and nintedanib remain the cornerstone of approved antifibrotic treatments, their clinical impact is generally disease-modifying rather than curative, and treatment is often limited by poor tolerance and systemic adverse effects (Antoniou et al., 2016; Petnak et al., 2021). Thus, there is an urgent need for novel therapeutic approaches that can effectively target key fibrogenic pathways while increasing drug exposure in diseased lung tissue.

Roxadustat (RXS) is a novel prolyl hydroxylase inhibitor (PHI) used clinically to treat anemia associated with chronic kidney disease. By reversibly inhibiting hypoxia-inducible factor (HIF), RXS alters the cellular adaptive response to hypoxia, thereby regulating genes involved in erythropoiesis and iron metabolism (Nasr et al., 2024; Saber et al., 2024). Beyond its hematopoietic activity, RXS has shown antifibrotic potential in experimental PF. Recently, in a bleomycin-induced PF model, RXS reduced the severity of lung damage by lowering levels of alpha-smooth muscle actin (α-SMA) and transforming growth factor beta 1 (TGF-β1), suggesting that RXS may inhibit the TGF-β1/Smad profibrotic pathway. (Huang et al., 2020; Zhang et al., 2026).

Although RXS has the potential to be effective in treating PF, it is limited by its pharmacokinetic and biodistribution characteristics. In a preclinical radiolabeled disposition study, RXS was distributed to multiple tissues, with relatively high levels in the liver, kidney, and lung within 1 h; however, the tissue-to-plasma radioactivity ratios were only 0.35–0.38 in lung, compared with 0.61–0.65 in liver and 0.61–0.66 in kidney, indicating that free RXS is not preferentially retained in lung tissue (Czock and Keller, 2022; Tsuruya et al., 2025). In addition, RXS is highly bound to plasma albumin (almost 99%) and has a blood-to-plasma ratio of about 0.6L (Czock and Keller, 2022). As a result, there is the potential for nonspecific systemic exposure to RXS, resulting in HIF-regulated response activity in non-target sites as well as clinically reported adverse events (hypertension, peripheral edema, hyperkalemia, thrombotic events, gastrointestinal disturbance, and seizure) (Li et al., 2023; Martínez-Miguel et al., 2025).

Beyond RXS-associated challenges, there are additional barriers associated with the pathological microenvironment of the fibrotic lung. PF is a mechanically stiff, collagen-rich, hypoxic, and immunologically remodeled alveolar microenvironment where injured epithelial cells, profibrotic macrophages, activated fibroblasts, and myofibroblasts support a self-perpetuating TGF-β/ECM loop (Tanabe et al., 2020; Moss et al., 2022). The consequences of dense ECM deposition, altered epithelial/endothelial barrier function, vascular remodeling, hypoxia, and chronic inflammation can compromise drug penetration, impair drug bioavailability at the site of therapy, and limit overall therapeutic effectiveness (Singh and Wairkar, 2024). Therefore, the therapeutic translation of PF could benefit from a lung-targeted delivery system that enhances drug localization within lung tissue, prolongs drug residence time, and minimizes nonspecific systemic exposure.

Silk fibroin nanoparticles (SKNPs) are an innovative biopolymeric nanocarrier platform designed for controlled drug delivery. SK is a naturally occurring protein-based biomaterial with high biocompatibility/biodegradability, good mechanical stability, and good chemical tunability (Gianak et al., 2019). SKNPs can protect incorporated drugs while enhancing their colloidal delivery via β-sheet-rich structural domains and providing a long-term release mechanism (Xu Z. et al., 2019). Additionally, SK–based carriers have also demonstrated functional anti-inflammatory activity via macrophage reprogramming, shifting M1 to M2 polarization and scavenging ROS, both of which are pathologically dysregulated in PF, providing a dual therapeutic and carrier function (Liu et al., 2025). Furthermore, SK proteins contain functional amino and carboxyl group side chains, facilitating drug loading and surface coating.

Gelatin (GT), a biodegradable polymer derived from collagen, offers additional benefits when used as a surface coating on SKNPs. GT is a biocompatible, hydrophilic, and amphoteric protein (Milano et al., 2023). It also contains high concentrations of functional amino, carboxyl, and hydroxyl groups, enabling modulation of surface charge, hydration, colloidal stability, and biological interactions (Shargh et al., 2017). Most importantly, GT can be degraded by gelatinases, such as matrix metalloproteinase-2 (MMP-2) and matrix metalloproteinase-9 (MMP-9). These enzymes have been reported to increase in the lungs of bleomycin-treated rats and in bronchoalveolar lavage fluid, primarily during early injury and remodeling (Kim et al., 2009; Inoue et al., 2023; Hamad et al., 2025). Therefore, coating SKNPs with GT may provide an enzyme-responsive interface that is susceptible to degradation in MMP-enriched fibrotic lung tissue, thereby facilitating RXS release at the diseased site and enhancing its therapeutic efficacy.

Based on this rationale, the objective of this study is to develop GT-coated RXS-SKNPs as a novel injectable biopolymeric nanoplatform for the treatment of PF. RXS-SKNPs were prepared by the desolvation method, followed by GT surface coating. The prepared formulations were evaluated *in vitro* for particle size (PS), polydispersity index (PDI), zeta potential (ZP), entrapment efficiency (%EE), release kinetics, and physical stability. Moreover, their morphological and structural characteristics were evaluated. *In vivo*, the optimized formulation was evaluated for lung distribution and therapeutic efficacy in a bleomycin-induced PF rat model. We hypothesized that the prepared GT-SKNPs would enhance the therapeutic efficacy of RXS in PF by providing an MMP-responsive GT coat that improves drug localization within lung tissues, thereby enhancing pulmonary exposure.

## 2 Materials and methods

### 2.1 Materials

Silk fibroin (SF; molecular weight 100–150 kDa), prepared from domesticated *Bombyx mori* silkworm, was obtained from the Key Laboratory of Advanced BioMatrix (5,930 Sea Lion Place, Carlsbad, CA, USA). Roxadustat (RXS; molecular formula C_19_H_16_N_2_O_5_; molecular weight 352.35 Da; purity ≥98% by HPLC) was purchased from Tocris Bioscience, Bio-Techne Ltd (Avonmouth, Bristol, United Kingdom). Type A gelatin, dimethyl sulfoxide (DMSO), methanol (HPLC grade), and genipin (purity 98%; molecular weight 226.23 Da) were purchased from Sigma-Aldrich (St. Louis, MO, USA). Recombinant human matrix metalloproteinase-2, MMP-2, gelatinase A, supplied as a 0.2 μm filtered solution in Tris, CaCl_2_, NaCl, and Brij-35, was purchased from R&D Systems, Minneapolis, MN, USA. p-Aminophenylmercuric acetate, APMA, was used for MMP-2 activation. Regenerated cellulose dialysis membranes with a molecular weight cut-off (MWCO) of 7 kDa and dimensions of 2.16 mm × 3.34 mm were supplied by FRY Scientific (Nashua, NH, USA). TNBSA reagent was purchased from Thermo Fisher Scientific (Waltham, MA, USA). Reagents used for buffer preparation, including sodium chloride (NaCl, ≥99%), anhydrous disodium hydrogen phosphate (Na_2_HPO_4_, 98%–100%), and anhydrous potassium dihydrogen phosphate (KH_2_PO_4_, 98%–100%), were sourced from Sigma-Aldrich (Steinheim, Germany). All other chemicals and solvents were of analytical grade and used as received unless otherwise stated.

### 2.2 Preparation of RXS-SKNPs

RXS-SKNPs were prepared using a desolvation-assisted nanoprecipitation method, based on a previously reported study (Moin et al., 2021). Briefly, 5 mL of RXS solution in DMSO (equivalent to 10 mg RXS) was added dropwise into an aqueous SF solution (5 mL, 0.25% w/v) under continuous magnetic stirring at 1,000 rpm at room temperature. Upon addition of the DMSO, RXS-SKNPs formed spontaneously due to desolvation. The resulting nano dispersion was stirred for an additional 15 min to ensure complete particle maturation, then centrifuged at 12,000 rpm for 30 min. The obtained pellet was washed three times with deionized water to remove residual DMSO (the supernatant after centrifugation and washing was collected to determine the free, non-entrapped drug concentration). After washing, the purified RXS-SKNPs were re-dispersed in deionized water, probe-sonicated at 30% amplitude for 10 min, and lyophilized to obtain dry RXS-SKNPs for further characterization and analysis. Blank SKNPs were prepared using the same methodology without RXS addition and used as a control formulation.

### 2.3 Gelatin coating of RXS-SKNPs

GT-RXS-SKNPs were prepared via electrostatic deposition of positively charged type A gelatin onto negatively charged RXS-SKNPs. Briefly, RXS-SKNPs were dispersed in PBS (pH 6.0) to obtain a final concentration of 1% w/v. Then, 5 mL of the RXS-SKNP dispersion was slowly added dropwise to 5 mL of the GT solutions at different concentrations (0.25, 0.5, 1.0, and 2.0% w/v) under mild magnetic stirring at room temperature. Stirring was continued for 20 min to allow the GT layer to adsorb onto the SKNP surface. The resulting coated NPs were centrifuged at 4,000 rpm for 30 min, and the collected particles were re-dispersed in 1% w/v genipin solution. The dispersion was gently stirred for an additional 10 min to induce genipin-mediated crosslinking of the GT layer on the NPs surface. Finally, the crosslinked GT-RXS-SKNPs were centrifuged again at 4,000 rpm for 30 min, separated, washed twice, and re-dispersed in deionized water for further characterization.

### 2.4 Characterization of the prepared NPs

#### 2.4.1 Size and surface charge determination

The mean PS, polydispersity index (PDI), and ZP of RXS-SKNPs and GT-RXS-SKNPs were determined by dynamic light scattering (DLS) and electrophoretic light scattering, respectively. Freshly prepared nanoparticle dispersions were diluted 1:10 with deionized water to avoid multiple scattering effects and then equilibrated at 25 °C before measurement. All measurements were performed in triplicate, and the results were reported as mean ± SD.

#### 2.4.2 Determination of entrapment efficiency (EE%)

The EE% of RXS-SKNPs and GT-RXS-SKNPs was determined after completion of the respective preparation, centrifugation, and washing steps. Briefly, for RXS-SKNPs, after centrifugation and washing, the wash fractions were combined with the original supernatant and analyzed for free RXS. RXS content was quantified using a validated spectrofluorimetric method at an emission wavelength (λem) of 406 nm after excitation (λex) at 260 nm. The method showed good linearity over the RXS concentration range of 0.1–100 μg/mL, with an *R* ^2^ of 0.9999. The EE% was calculated using the following equation:

$$EE\;\left(\%\right)=\frac{W_{i}-W_{f}}{W_{i}}\times 100$$

Where *W*_*i*_ denotes the initial RXS weight added, and *W*_*f*_ denotes the free RXS amount.

Moreover, to assess whether any drug loss occurred during the GT coating process, all supernatants and washing fractions collected during the coating and purification steps were analyzed for free RXS content.

#### 2.4.3 Transmission electron microscopy (TEM)

The morphological characteristics of the prepared RXS-SKNPs and GT-RXS-SKNPs were evaluated using TEM (JEM-2100, JEOL Ltd., Japan). One drop of the diluted dispersion was deposited onto a carbon-coated copper grid and left for 2 min to allow NPs attachment. The excess dispersion was gently removed with filter paper. Negative staining was performed by adding 1–2 drops of 1% phosphotungstic acid solution to the grid for 30–60 s, after which the excess stain was carefully blotted off. The grid was then air-dried at room temperature before TEM examination.

#### 2.4.4 Fourier transformation infrared (FTIR)

The FTIR spectra of RXS, GT, blank SKNPs, and GT-RXS-SKNPs were examined by the KBr pellet method using a spectrophotometer (Shimadzu, model 8,300, Japan). Samples were finely ground, and approximately 4 mg of each was thoroughly mixed with KBr. FTIR spectra were recorded over the wavenumber range of 4,000–500 cm^-1^.

#### 2.4.5 Surface-accessible amino group assay

Surface-accessible primary amino groups on the NPs were evaluated using a TNBSA colorimetric assay following a previous method (Wang et al., 2017). Briefly, blank SKNPs, RXS-SKNPs, blank GT-SKNPs, and RXS-GT-SKNPs were centrifuged and washed three times with deionized water. The washed NPs pellets were redispersed in 0.1 M NaHCO3 buffer, pH 8.5, to obtain a final NPs concentration of 1 mg/mL. For the assay, 500 µL of each dispersion was mixed with 250 µL of TNBSA reagent and incubated at 37 °C for 2 h in the dark. After incubation, 250 µL of 10% SDS was added, followed by 125 µL of 1 N HCl to stop the reaction. The samples were then centrifuged to remove suspended NPs, and the absorbance of the clear supernatant was measured at 335 nm (Wang et al., 2017). To correct for background absorbance, sample blanks were prepared by treating each dispersion under the same conditions but without adding TNBSA. A reagent blank containing buffer and TNBSA without NPs was also included. The corrected absorbance was calculated according to the following equation:

$$A_{corrected}=A_{Sample\;with\;TNBSA}-A_{Sample\;without\;TNBSA}-A_{reagent\;blank}$$

### 2.5 MMP-2-triggered colloidal remodeling study

The response of GT-RXS-SKNPs and uncoated RXS-SKNPs to activated MMP-2 was evaluated by monitoring changes in hydrodynamic particle size. Recombinant human pro-MMP-2 was diluted to 100 μg/mL in MMP assay buffer containing 50 mM Tris-HCl, 10 mM CaCl_2_, 150 mM NaCl, and 0.05% w/v Brij-35, pH 7.5. APMA, prepared as a 100 mM stock solution in DMSO, was added to obtain a final APMA concentration of 1 mM. The enzyme solution was incubated at 37 °C for 1 h to activate pro-MMP-2 and was subsequently diluted with assay buffer to the required working concentrations. 1 mL of each dispersion was mixed with 5 mL of activated MMP-2 working solution to produce final MMP-2 concentrations of 50, 100, or 300 ng/mL in a total incubation volume of 6 mL. Corresponding enzyme-free controls were prepared by mixing the nanoparticle dispersions with an equal volume of MMP assay buffer without MMP-2. Samples were incubated at 37 °C under gentle agitation. At 4, 12, and 24 h, aliquots were collected and analyzed by dynamic light scattering under the same measurement conditions used for the initial particle-size determination. Measurements were performed using three independently prepared samples, and the results were expressed as mean ± SD.

### 2.6 *In vitro* MMP-2-triggered release study

*In vitro* release study of uncoated RXS-SKNPs and GT-coated RXS-SKNPs was performed in MMP-2 incorporated and MMP-2 free media by dialysis bag method. The activated enzyme was diluted in MMP assay buffer (50 mM Tris-HCl, 10 mM CaCl2, 150 mM NaCl, 0.05% w/v Brij-35, pH 7.4). Then, 1 mL of each formulation (equivalent to 1 mg RXS) was added to a cellulose dialysis bag. In the enzyme-treated group, 1 mL of activated MMP-2 working solution (600 ng/mL) was added to the formulation in the bag, resulting in a final donor volume of 2 mL and a final MMP-2 concentration of 300 ng/mL. For the enzyme-free control groups, the formulation was mixed with an equal volume of MMP assay buffer without MMP-2. The dialysis bags were then tightly sealed and placed in 50 mL of the release medium maintained at 37 °C ± 0.5 °C with stirring at 100 RPM. At specific time points (1, 2, 3, 4, 5, 6, 12, and 24 h), aliquots were removed and replaced with an equal volume of fresh medium to maintain sink conditions. The amount of released RXS was determined by a validated spectrofluorimetric method (RF-6000 model, Shimadzu, Tokyo, Japan) at λem 406 nm and λex 260 nm. Moreover, the release data for GT-coated RXS-SKNPs were fitted to zero-order, first-order, Higuchi, and Korsmeyer-Peppas models to mathematically examine the release kinetics, using the DDsolver add-in for Microsoft Excel (Zhang et al., 2010b). The degree of correlation was used to determine which mathematical model was best.

### 2.7 Stability study

The stability of the optimized formulation, GT-RXS-SKNPs, was evaluated during 3-month short-term storage at two temperatures: room temperature (25 °C ± 2 °C) and refrigerated temperature (4 °C ± 1 °C). The freshly prepared nanoparticle dispersion was added into sealed glass vials and stored under the selected conditions. Samples were withdrawn at 0, 1, 2, and 3 months to evaluate physicochemical stability. At each point, samples were visually examined for any sign of aggregation, precipitation, or phase separation. The PS, PDI, ZP, and EE% were determined at each time point by the same validated method described for the freshly prepared formulation.

### 2.8 *In vitro* hemolysis study

The hemocompatibility of GT-RXS-SKNPs was evaluated using an *in vitro* hemolysis assay (Peng et al., 2024). Fresh blood was collected from male Wistar rats into heparinized tubes. The blood was centrifuged at 4,000 rpm for 10 min to separate the plasma, and the packed red blood cells were collected. The erythrocyte pellet was washed with normal saline by repeated resuspension and centrifugation at 4,000 rpm for 10 min. The washed RBCs were then diluted with PBS to prepare a 2% v/v erythrocyte suspension. A stock dispersion of GT-RXS-SKNPs was diluted with PBS to obtain SKNP-equivalent concentrations of 0.05, 0.125, 0.25, 0.5, 1.25, and 2.5 mg/mL. For each concentration, the nanoparticle dilution was mixed with the erythrocyte suspension at a 4:1 volume ratio. Briefly, 800 µL of each nanoparticle dilution was mixed with 200 µL of the 2% erythrocyte suspension, yielding a final incubation volume of 1 mL per tube. After incubation for 3 h at 37 °C, the tubes were centrifuged at 4,000 rpm for 10 min to sediment intact erythrocytes and nanoparticle residues. The supernatant was carefully collected, and the absorbance was measured spectrophotometrically at 540 nm. Distilled water and normal saline were used as positive and negative controls, respectively. The percentage hemolysis was calculated using the following equation:

$$Hemolysis\left(\%\right)=\frac{A_{sample}-A_{negative\;control}}{A_{positive\;control}-A_{negative\;control}}\times 100$$

### 2.9 Biological evaluation

#### 2.9.1 Animals

Adult male Wistar rats weighing 190–200 g were used in the *in vivo* lung distribution and in the therapeutic efficacy experiment. Animals were obtained from Theodor Bilharz Research Institute and were housed in polypropylene cages under standard laboratory conditions of 21 °C ± 2 °C, 45%–55% relative humidity, and a 12 h light/12 h dark cycle, with free access to standard rodent chow and water. Rats were acclimatized for at least 7 days before the experiment.

All experimental procedures were approved by the Institutional Animal Care and Use Committee at Delta University for Science and Technology (approval number: FPDU3520/3C) and were performed in accordance with the ARRIVE guidelines, EU Directive 2010/63 on the protection of animals used for scientific purposes as well as relevant institutional and national regulations for animal care and use. Animals were monitored daily for body weight, respiratory distress, activity, grooming, food and water intake, signs of pain, and morbidity. Humane endpoints included severe or persistent dyspnea, inability to reach food or water, moribund behavior, marked hypothermia, non-resolving bleeding, infection at the surgical site, or more than 20% body-weight loss.

#### 2.9.2 *In vivo* lung distribution study

Male Wistar rats with PF were randomly divided into three treatment groups (n = 15 per group). The three groups received a single IV administration of free aqueous RXS solution, uncoated RXS-SKNPs, or GT-RXS-SKNPs (both dispersed in PBS, pH 7.4), all at a dose equivalent to 5 mg/kg RXS. At predetermined time points of 1, 3, 6, 12, and 24 h after administration, rats were deeply anesthetized by intraperitoneal injection of ketamine/xylazine (ketamine 80 mg/kg and xylazine 10 mg/kg, i. p.) until loss of pedal withdrawal reflex. Blood samples were then collected from three rats per group via cardiac puncture, and animals were euthanized by exsanguination under deep anesthesia. Death was confirmed by cessation of respiration and heartbeat before lung excision. Samples were transferred into heparinized tubes and centrifuged at 3,000 rpm for 15 min to separate plasma. The obtained plasma samples were stored at −80 °C until RXS quantification. The isolated tissues were gently rinsed with normal saline to remove surface blood, blotted dry with filter paper, accurately weighed, and homogenized in acetonitrile using an ultra-homogenizer (T25 digital ULTRA-TURRAX®, IKA-Werke GmbH and Co. KG, Germany). The final volume of each lung homogenate was recorded, and the homogenized samples were stored at −80 °C until analysis. A destructive-sampling design was used, with three independent rats assigned to each sampling time point. Plasma and lung RXS concentrations were expressed as mean ± SD at each time point. Composite mean concentration–time profiles were constructed for each treatment group and analyzed by noncompartmental analysis using PKSolver Software (Zhang et al., 2010a). Different kinetic parameters, including *AUC*_*0*_ *–*_*24*_, *C*_max_, *t*_max_, *t*_*1/2*_, and *MRT*, were calculated following non-compartmental analysis.

To quantitatively evaluate pulmonary targeting performance, several pharmacokinetic indices were calculated. The apparent lung-to-plasma partition coefficient *Kp* was calculated by comparing lung concentration (*C*_*lung*_) to plasma concentration (*C*_*plasma*_) at each time point, using the following equation:

$$K_{p}=\frac{C_{lung}}{C_{plasma}}$$

Additionally, the relative lung concentration efficiency (*RCe*), the relative systemic exposure (*RSe*), and the drug distribution index (*DDI*) were also calculated using the following equations:

$$RCe=\frac{AUC_{0-24,lung,test\;}}{AUC_{0-24,lung,reference}}$$

$$RSe=\frac{AUC_{0-24,plasma,test\;}}{AUC_{0-24,plasma,reference}}$$

$$DDI=\frac{RCe}{RSe}$$

Where reference stands for the free RXS or uncoated-RXS-SKNPs group, and test refers to uncoated RXS-SKNPs or GT-coated SKNPs, depending on the comparison type. These indices indicate the comparative RXS distribution into the lung relative to systemic exposure. *RCe* and *DDI* values >1 and RSe values <1 indicate improved pulmonary delivery relative to systemic exposure, which is favorable for lung-selective delivery.

#### 2.9.3 RXS analysis

RXS concentrations in plasma and lung homogenates were quantified using a spectrofluorimeter (RF-6000 model, Shimadzu, Tokyo, Japan). Calibration curves were prepared by spiking blank plasma and blank lung homogenate with RXS working solutions at different concentrations. The samples were extracted and deproteinized using methanol and acetonitrile (1:1 v/v) as organic solvents. After thorough vortex mixing, the samples were centrifuged at 3,000 rpm for 15 min. The clear supernatant was then collected, filtered (0.22 µm), evaporated, and the residue was dissolved in methanol before spectrofluorimetric measurement.

The fluorescence intensity of RXS was measured at λem of 406 nm and at λex of 260 nm. The method showed good linearity over the RXS concentration range of 0.1–100 μg/mL in both plasma and lung homogenate matrices, with *R* ^*2*^ values of 0.9997 and 0.9995, respectively. For plasma, the LOD and LOQ were 0.0249 μg/mL and 0.0756 μg/mL, respectively. For lung homogenate, the LOD and LOQ were 0.0325 μg/mL and 0.0926 μg/mL, respectively. The mean recovery values were 100.25% for plasma and 98.66% for lung homogenate. The method also showed acceptable precision, with %RSD values less than 2% for both matrices. Blank plasma and blank lung homogenate samples were processed using the same procedure to correct for possible matrix interference.

#### 2.9.4 Therapeutic efficacy study in bleomycin-induced pulmonary fibrosis

##### 2.9.4.1 Materials used in therapeutic efficacy study

Bleomycin was freshly dissolved in sterile PBS (pH 7.4) immediately before intratracheal instillation. The main reagents, ELISA kits, antibodies, and assay-specific applications used for the biological evaluation are summarized in Table 1.

**TABLE 1:** Key reagents, commercial assay kits, antibodies, and their applications in the therapeutic efficacy study.

Endpoint/reagent	Supplier/catalog number	Sample/assay use
Bleomycin	Bleocel 15 mg/vial; cell pharm GmbH	Single intratracheal induction of pulmonary fibrosis
Masson trichrome stain	Sigma-aldrich/Merck	Collagen histochemistry and collagen-positive area quantification
Hydroxyproline assay	Sigma-aldrich/Merck, MAK008	Colorimetric lung collagen surrogate; absorbance at 560 nm
TNF-α ELISA	R&D systems, RTA00	BALF TNF-α concentration
IL-6 ELISA	R&D systems, R6000B	BALF IL-6 concentration
IL-1β ELISA	R&D systems, RLB00	BALF IL-1β concentration
TGF-β1 ELISA	R&D systems, DB100C	Total TGF-β1 in acid-activated lung tissue homogenate
Total MMP-2 ELISA	R&D systems, MMP200	Total MMP-2 in lung tissue homogenate
Rat total MMP-9 ELISA	R&D systems, RMP900	Total MMP-9 in lung tissue homogenate
α-SMA antibody	Cell signaling technology, #19245	IHC detection of myofibroblast activation
HRP/DAB detection system	Dako/Agilent EnVision system	IHC chromogenic visualization
Total protein assay	BCA; thermo Fisher scientific	Normalization of lung homogenate analytes

##### 2.9.4.2 Experimental design and treatment protocol

Rats were randomly allocated into five experimental groups, with eight biological replicates per group included in the final analysis (n = 8 rats/group). Group 1 served as the normal control group and received intratracheal PBS/saline followed by intravenous vehicle. Group 2 served as the bleomycin/PF control group and received intratracheal bleomycin followed by intravenous vehicle. Group 3 received intratracheal bleomycin followed by free RXS. Group 4 received intratracheal bleomycin followed by uncoated RXS-SKNPs. Group 5 received intratracheal bleomycin followed by GT-RXS-SKNPs.

All RXS-containing treatments were administered at an equivalent RXS dose of 5 mg/kg by slow intravenous injection through the lateral tail vein. Free RXS, RXS-SKNPs, and GT-RXS-SKNPs were administered every 48 h from day 7 after bleomycin instillation until day 27. The injection volume was kept constant across groups. On day 28, rats were deeply anesthetized with ketamine/xylazine (ketamine 80 mg/kg and xylazine 10 mg/kg, i. p.) until loss of pedal withdrawal reflex for terminal BALF collection and lung tissue harvesting. After BALF collection, animals were euthanized by exsanguination via cardiac puncture under deep anesthesia, and death was confirmed by cessation of respiration and heartbeat before tissue collection. The delayed-start protocol was used to evaluate therapeutic activity during established post-bleomycin inflammatory-to-fibrotic remodeling rather than a purely preventive effect.

##### 2.9.4.3 Induction of bleomycin-induced pulmonary fibrosis

Pulmonary fibrosis was induced on day 0 by a single intratracheal instillation of bleomycin. Rats were anesthetized by intraperitoneal injection of ketamine/xylazine mixture, ketamine 80 mg/kg and xylazine 10 mg/kg, to achieve a surgical plane of anesthesia before intratracheal instillation. After the absence of pedal withdrawal reflex was confirmed, the ventral neck region was shaved and disinfected. A small midline cervical incision was made to expose the trachea. Bleomycin solution was slowly instilled into the trachea at a dose of 5 mg/kg in sterile PBS, using a final instillation volume of 0.5 mL/kg. Normal control rats underwent the same anesthesia and sham surgical procedure and received an equal volume of sterile PBS intratracheally.

Immediately after instillation, rats were held in a near-vertical position and gently rotated for approximately 30–60 s to promote pulmonary distribution of the instilled solution. The incision was closed using sterile sutures, the wound area was disinfected, and animals were kept warm until complete recovery. Post-procedure care were provided according to local veterinary guidance and the approved animal protocol.

##### 2.9.4.4 Rationale for dose, route, treatment timing, and omission of direct HIF-pathway endpoints

A single intratracheal bleomycin dose of 5 mg/kg was selected because this dose produces reproducible fibrotic remodeling, collagen deposition, increased hydroxyproline content, BALF inflammation, and histopathological injury by approximately day 21–28 in rat pulmonary fibrosis protocols (Tukhovskaya et al., 2025). The 5 mg/kg RXS-equivalent dose was selected to match the completed lung-distribution study, in which free RXS, RXS-SKNPs, and GT-RXS-SKNPs were compared at the same RXS-equivalent dose. The intravenous route was retained because the optimized formulation was designed and characterized as an injectable nanoplatform, and this route allowed direct continuity between the biodistribution and efficacy studies. Treatment was started on day 7 to avoid overemphasizing acute prophylactic anti-inflammatory effects and to support a therapeutic antifibrotic claim.

Direct HIF-biology endpoints, including HIF-1α, PHD2, VEGF as a HIF target, EPO, or broad HIF-responsive gene panels, were not assessed in this efficacy arm. This choice was made to keep the study focused on delivery-driven antifibrotic efficacy rather than a dedicated HIF-mechanism analysis. Because HIF signaling in pulmonary fibrosis is highly context dependent and may vary according to disease stage, cell type, oxygen tension, and sampling time, rigorous mechanistic interpretation would require dedicated time-course sampling, validated HIF-pathway readouts, and additional pathway-specific controls (Shimoda and Semenza, 2011). Therefore, mechanistic interpretation was limited to the observed changes in fibrosis, inflammation, TGF-β1, matrix remodeling markers, and myofibroblast activation.

##### 2.9.4.5 BALF collection and processing

On day 28, rats were deeply anesthetized by intraperitoneal injection of ketamine/xylazine (ketamine 80 mg/kg and xylazine 10 mg/kg, i. p.) until loss of pedal withdrawal reflex, and the trachea was cannulated *in situ*. BALF was collected by lavaging the lung three times with 1 mL aliquots of chilled sterile PBS. During each lavage, the fluid was slowly instilled and gently withdrawn. After BALF collection, animals were euthanized by exsanguination via cardiac puncture under deep anesthesia, and death was confirmed by cessation of respiration and heartbeat before lung tissue harvesting. BALF recovery was recorded for each animal. BALF samples were centrifuged at 2000 rpm for 10 min at 4 °C. The cell-free supernatants were aliquoted and stored at −80 °C until ELISA assessment of TNF-α, IL-6, and IL-1β. BALF cytokine concentrations were expressed as pg/mL BALF.

##### 2.9.4.6 Lung tissue collection and homogenate preparation

After BALF collection, the lungs were carefully excised, rinsed with ice-cold PBS to remove surface blood, and blotted dry. Lung samples assigned for histology and immunohistochemistry were fixed in 10% neutral-buffered formalin for 24 h and processed for paraffin embedding. Lung samples assigned for biochemical assays were snap-frozen and stored at −80 °C until analysis. Lung tissue homogenates were prepared in ice-cold PBS or Tris-HCl/EDTA buffer containing protease inhibitors where required. Homogenates were centrifuged at 5,000 × g for 10 min at 4 °C, and the supernatants were stored at −80 °C. Total protein concentration was determined using a BCA assay, and lung homogenate analytes were normalized to total protein.

##### 2.9.4.7 H&E staining and H&E-based histopathological scoring

Formalin-fixed lung samples were dehydrated through ascending grades of ethanol, cleared in xylene, embedded in paraffin, sectioned at 4–5 µm thickness, and mounted on glass slides. Sections were deparaffinized, rehydrated, and stained with hematoxylin and eosin (H&E). Lung architecture, alveolar wall thickening, inflammatory-cell infiltration, consolidation, alveolar collapse, epithelial injury, hemorrhage, and fibrotic distortion were examined by a blinded observer.

H&E sections were semi-quantitatively scored using a composite histopathological score ranging from 0 to 15.10 randomly selected non-overlapping parenchymal fields were examined per animal. Five histological features were graded in each field: alveolar septal thickening, inflammatory-cell infiltration, alveolar collapse/architectural distortion, edema/hemorrhage/exudation, and epithelial injury/consolidation. Each feature was assigned a score of 0, absent; 1, mild; 2, moderate; or 3, severe. The sum of the five parameters yielded a field score from 0 to 15. The mean of the 10 field scores was calculated to obtain one final H&E score per animal. The animal, not the microscopic field, was considered the biological replicate.

##### 2.9.4.8 Modified ashcroft scoring of pulmonary fibrosis

Pulmonary fibrosis severity was quantified using the modified Ashcroft scoring system. Ten randomly selected non-overlapping lung parenchymal fields were examined per animal at fixed magnification while avoiding large bronchi, major blood vessels, pleural edges, folded tissue, and processing artifacts. Each field was assigned a score from 0 to 8, where 0 indicated normal lung, 1 indicated minimal fibrous thickening of alveolar or bronchiolar walls, 2-3 indicated moderate wall thickening without obvious architectural damage, 4-5 indicated increased fibrosis with definite architectural distortion, 6-7 indicated severe fibrotic distortion with large fibrous areas or honeycomb-like changes, and 8 indicated total fibrous obliteration of the field. The mean of the 10 field scores was calculated to obtain one final Ashcroft score per animal.

##### 2.9.4.9 Masson trichrome staining and collagen-positive area quantification

Collagen deposition was assessed by Masson trichrome staining. Paraffin-embedded lung sections were deparaffinized, rehydrated, stained according to the kit protocol, dehydrated, cleared, and mounted. Representative images were acquired under identical microscope and acquisition settings by a blinded investigator. Collagen fibers were identified by blue staining.

Quantitative morphometry was performed using ImageJ software (NIH, Bethesda, MD, USA). Ten randomly selected non-overlapping lung parenchymal fields were analyzed per animal. Large bronchi, major blood vessels, pleural edges, tissue folds, empty spaces, and staining artifacts were excluded. The blue-stained collagen area was thresholded using a fixed color-threshold setting applied uniformly across all groups. Collagen deposition was expressed as Masson collagen-positive area (%) = collagen-positive area/total analyzed tissue area × 100.

##### 2.9.4.10 Immunohistochemical detection and quantification of α-SMA

Immunohistochemistry was performed on 4–5 µm paraffin-embedded lung sections to assess myofibroblast activation. Sections were deparaffinized in xylene, rehydrated through descending alcohol concentrations, and subjected to heat-mediated antigen retrieval using citrate buffer (10 mM, pH 6.0). Endogenous peroxidase activity was blocked with 0.3% hydrogen peroxide, and nonspecific binding was reduced using normal serum.

Sections were incubated overnight at 4 °C with a primary antibody against α-SMA (Cell Signaling Technology, #19245; dilution 1:250). Slides were then incubated with an HRP-labeled polymer detection system, and diaminobenzidine (DAB) was used as the chromogen. Sections were counterstained with Mayer’s hematoxylin, dehydrated, cleared, and mounted. Negative controls were processed by omitting the primary antibody.

α-SMA immunostaining was quantified by a blinded investigator using ImageJ. Ten randomly selected non-overlapping parenchymal fields were analyzed per animal using a fixed threshold applied uniformly across all groups. α-SMA expression was expressed as α-SMA-positive area (%) = α-SMA-positive DAB-stained area/total analyzed tissue area × 100.

##### 2.9.4.11 Determination of lung hydroxyproline content

Lung hydroxyproline content was measured as a quantitative surrogate of total collagen burden using a colorimetric hydroxyproline assay kit (Sigma-Aldrich/Merck, MAK008). Weighed lung tissue samples were hydrolyzed according to the kit protocol. Aliquots of hydrolysate were reacted with oxidation reagent followed by DMAB/Ehrlich reagent, and absorbance was measured at 560 nm using a calibrated microplate reader. Hydroxyproline concentrations were calculated from a standard curve. Hydroxyproline content was expressed as µg/mg protein. Each biological sample was assayed in duplicate, and the duplicate mean was used for statistical analysis.

##### 2.9.4.12 ELISA assessment of BALF cytokines and lung tissue profibrotic/gelatinase markers

BALF concentrations of TNF-α, IL-6, and IL-1β were measured using ELISA: TNF-α Quantikine ELISA Kit (R&D Systems, RTA00), IL-6 Quantikine ELISA Kit (R&D Systems, R6000B), and IL-1β Quantikine ELISA Kit (R&D Systems, RLB00), respectively. BALF samples and standards were assayed in duplicate according to the manufacturer’s protocols. Cytokine concentrations were expressed as pg/mL BALF.

Total TGF-β1 was measured in lung tissue homogenates using TGF-β1 Quantikine ELISA Kit (R&D Systems, DB100C). Lung TGF-β1 concentrations were normalized to total protein and expressed as pg/mg protein.

Total MMP-2 and total MMP-9 were measured in lung tissue homogenates using the Total MMP-2 Quantikine ELISA Kit (R&D Systems, MMP200) and the Total MMP-9 Quantikine ELISA Kit (R&D Systems, RMP900), respectively. These assays quantify total gelatinase abundance, including pro-, active, and inhibitor-complexed forms. Results were normalized to total protein and expressed as ng/mg protein. All samples and standards were assayed in duplicate, and the duplicate mean was used for statistical analysis.

##### 2.9.4.13 Statistical analysis

For all terminal endpoints, n = 8 rats per group were used as biological replicates. For histological scoring, Masson morphometry, and α-SMA immunohistochemistry, 10 non-overlapping microscopic fields were analyzed per animal. These fields were treated as within-animal technical/sampling fields and were averaged to generate one value per animal. For ELISA and hydroxyproline measurements, each biological sample was assayed in duplicate technical wells, and the duplicate mean was used as one biological value. Statistical comparisons were performed using animal-level values only. Data were analyzed using GraphPad Prism version 9. Continuous parametric data were expressed as mean ± SD and analyzed using one-way ANOVA followed by Tukey’s multiple-comparison test when normality and variance assumptions were acceptable. Ordinal histological scores, including the H&E histopathological score and modified Ashcroft score, were analyzed using the Kruskal–Wallis test followed by Dunn’s multiple-comparison test. Predefined pairwise comparisons among treatment groups were interpreted according to the multiplicity-adjusted *post hoc* results. Differences were considered statistically significant at p < 0.05. In figures, ns indicated non-significance, *p < 0.05, **p < 0.01, ***p < 0.001, and ****p < 0.0001.

## 3 Results

### 3.1 Preparation and characterization of RXS-SKNPs and GT-coated RXS-SKNPs

The preparation conditions for RXS-SKNPs were established during preliminary formulation trials. RXS was dissolved in DMSO and added to an equal volume of aqueous SF solution, resulting in a DMSO-to-aqueous phase ratio of 1:1 v/v. Under these conditions, DMSO acted as a desolvating solvent, reducing SF hydration and promoting the molecular rearrangement and self-assembly of SF into nanoparticles. The SF concentration was selected based on preliminary formulation screening, as summarized in Supplementary Table S1. Although 0.125% w/v SF produced smaller nanoparticles, it resulted in markedly low RXS entrapment efficiency, indicating insufficient polymeric matrix capacity for drug incorporation. Increasing SF concentration to 0.25% w/v substantially improved EE% while maintaining homogeneous nanoparticles with suitable nanoscale size, acceptable PDI, and stable negative surface charge. In contrast, 0.5% w/v SF produced larger and more polydisperse nanoparticles, while 1% w/v SF resulted in phase separation (Perteghella and Bari, 2020). Therefore, 0.25% w/v SF was selected as the optimal concentration for subsequent gelatin-coating studies because it provided the most appropriate balance between particle size, colloidal stability, and drug entrapment.

The coating process is designed based on the electrostatic interaction between positively charged GT and negatively charged SKNPs. The interaction medium was chosen to be at pH 6.0, as the isoelectric point (IEP) of GT (Type A) is around pH 7–9 (De Farias et al., 2023), whereas SK has an IEP around pH 4–5 (Škrbić et al., 2024); accordingly, at this pH, GT is expected to remain positively charged while SK remains negatively charged, enabling electrostatic coating. Moreover, various GT concentrations, ranging from 0.25% to 2% w/v, were examined to modulate coating density and surface charge. Our aim was to get slightly negative surfaces, since NPs with near-neutral or slightly negative surfaces are generally preferred to reduce rapid plasma protein adsorption, opsonization, and clearance (Aggarwal et al., 2009; Hamad et al., 2026). The screening was intentionally started at a low gelatin concentration of 0.25% w/v to obtain a thin gelatin layer while avoiding excessive positive surface charge.

The physicochemical characterization results of the prepared RXS-SKNPs and GT-RXS-SKNPs are summarized in Table 2. Uncoated RXS-SKNPs (F1) exhibited a PS of 145.72 ± 2.74 nm and a PDI of 0.253 ± 0.031, indicating a homogeneous formulation with a narrow particle distribution. Additionally, RXS-SKNPs exhibited a relatively high EE% of 73.59% ± 1.25%. This may be related to the hydrophobic nature of RXS, which could favor its incorporation into SK’s bulk hydrophobic domains through hydrophobic interactions. SK possesses alternating hydrophobic and hydrophilic domains, with hydrophobic β-sheet-forming regions that can interact with poorly soluble or hydrophobic drug molecules (Pham and Tiyaboonchai, 2020). The ZP value of RXS-SKNPs was −30.28 ± 1.01. The negative charge is expected because SF contains acidic amino acid residues, mainly aspartic acid and glutamic acid, with carboxyl groups that can ionize to (-COO^-^) in aqueous media (Niu et al., 2021).

**TABLE 2:** Physicochemical characterization of RXS-SKNPs before and after coating with different GT concentrations (0.25%–2% w/v).

Formula	GT (% w/v)	Appearance	PS (nm)	PDI	ZP (mV)	EE (%)
F1	0	Homogenous	145.72 ± 2.74	0.253 ± 0.031	−30.28 ± 1.01	73.59 ± 1.25
F2	0.25	Homogenous	165.43 ± 4.02	0.304 ± 0.007	−19.07 ± 1.46	71.37 ± 1.08
F3	0.5	Homogenous	194.26 ± 2.94	0.335 ± 0.009	−15.19 ± 2.38	72.11 ± 0.81
F4^a^	1	Precipitated	-	-	-	-
F5	2	Homogenous	232.42 ± 4.21	0.319 ± 0.008	18.31 ± 1.37	72.43 ± 0.71

^a^ F4 was excluded from further characterization because the addition of RXS-SKNPs, into 1% w/v GT, solution caused immediate precipitation.

Data are expressed as mean ± SD, n = 3.

Gelatin coating was optimized by screening different GT concentrations, as presented in Table 2. Since the RXS-SKNP dispersion used for coating was 1% w/v, 0.25% w/v GT was selected as the lowest practical coating concentration, corresponding to an estimated GT:RXS-SKNP ratio of 0.25:1. This low initial ratio was expected to provide partial surface coverage without excessive charge neutralization or conversion to a positively charged surface. This was supported experimentally by the slight reduction in the negative ZP of RXS-SKNPs after coating with 0.25% GT, indicating surface interaction while maintaining a negative surface charge. Increasing GT concentration to 0.5% w/v produced a more evident increase in PS and a further shift of ZP toward less negative values, while maintaining a homogeneous appearance, acceptable PDI, and preserved EE%. In contrast, higher GT concentrations caused unfavorable colloidal behavior, including precipitation and/or excessive alteration of surface charge.

Increasing the GT percentage from 0.25% to 2% w/v significantly increased the mean PS from 165.43 ± 4.02 (F2) to 232.42 ± 4.21 (F5), indicating deposition of the GT layer around the SKNP surface. Also, there was a significant reduction in the ZP value from −19.07 ± 1.46 mV (F2) to −15.19 ± 2.38 mV (F3), while 2% w/v GT (F5) caused a charge reversal into the positive direction with a ZP value of +18.31 ± 1.37 mV. The gradual charge reduction and charge reversal indicate partial shielding of the anionic SKNPs surface by cationic type A GT. Noting that, using 1% w/v GT (F4) led to immediate precipitation, likely due to insufficient increase in the positive charge on SKNPs at this GT percentage, resulting in poor stability and particle aggregation. This behavior was also observed in a previous study, where coating chitosan NPs with alginate at a concentration below 3% resulted in an immediate precipitation (Elmorsy et al., 2025).

Moreover, a non-significant reduction in the EE% was observed after GT coating, indicating a minimal drug loss during the coating process. A similar behavior after polymeric coating has been reported previously (Elmorsy et al., 2025; Hamad et al., 2025). However, in these studies, this loss was significant. In contrast, the non-significant decrease observed in the present study suggests that RXS was strongly retained within the SKNP matrix. This behavior may be attributed to the compact, structurally stable nature of SKNPs, particularly their β-sheet-rich protein network, which can provide a rigid matrix that limits premature drug diffusion and leakage.

**FIGURE 1:**
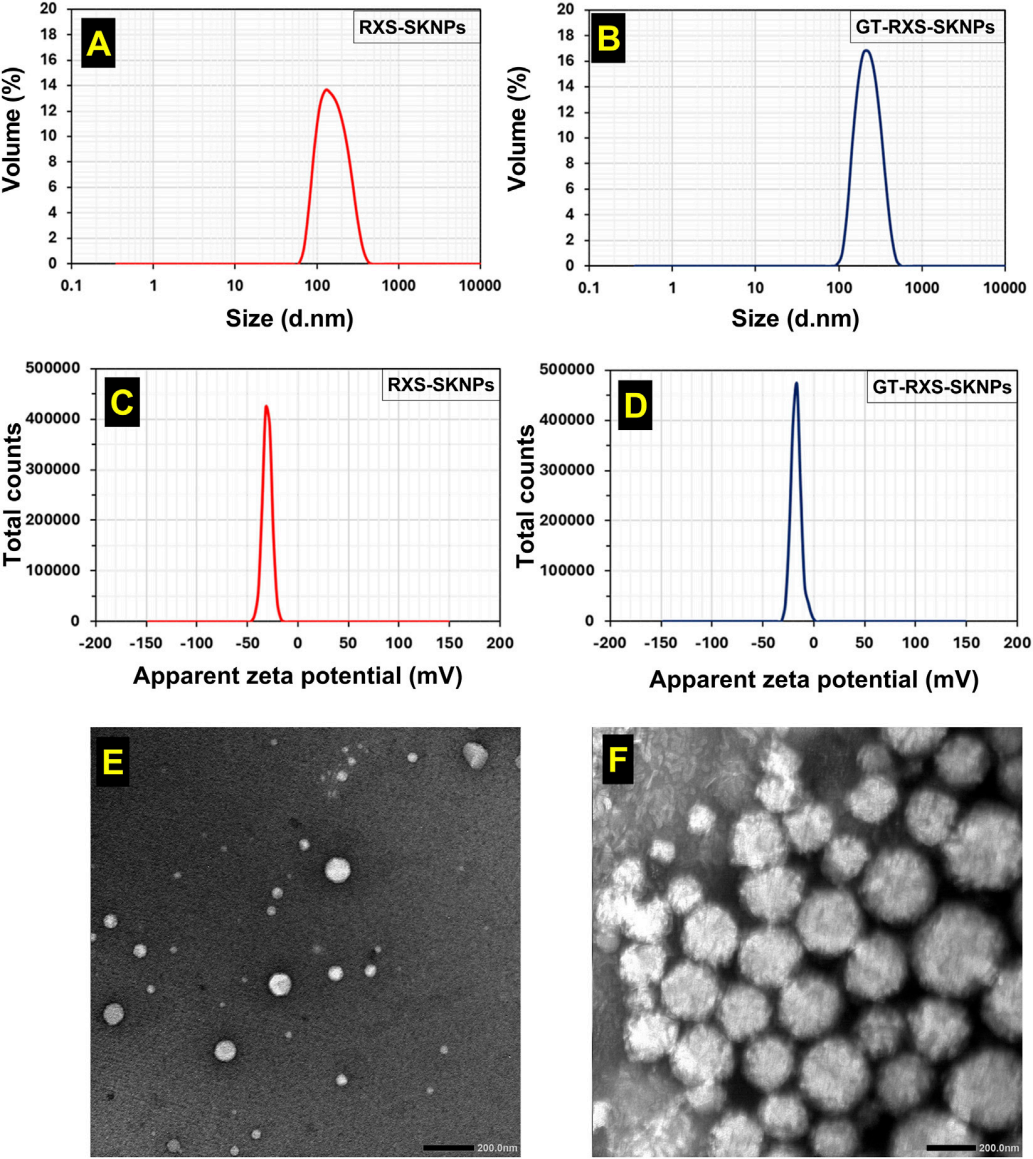
Particle size distribution of uncoated RXS-SKNPs **(A)** and GT-RXS-SKNPs **(B)**. Zeta potential distribution of uncoated RXS-SKNPs **(C)** and GT-RXS-SKNPs **(D)**. TEM image of uncoated RXS-SKNPs **(E)** and optimized GT-RXS-SKNPs **(F)**.

Based on these results, F3 (0.5% w/v GT) was selected as the optimized GT-RXS-SKNPs. This selection was based on the aim of achieving a slightly negative charge. The moderately negative ZP of F3 was considered preferable to the positive charge observed for F5, as highly cationic surfaces may promote stronger nonspecific interactions with biological components, including serum proteins and cell membranes, thereby increasing aggregation tendency and biological clearance. Although F5 showed a comparable EE% and slightly lower PDI than F3, it exhibited a larger PS, making it less suitable for further development. Compared with F2, F3 showed a less negative ZP and a slightly higher EE%, along with modest increases in PS and PDI. Therefore, F3 was considered the most suitable formulation for further studies. Figure 1A,B show the mean PS of uncoated RXS-SKNPs and optimized GT-RXS-SKNPs (F3), respectively, while Figures 1C,D show their corresponding ZP distributions.

Figures 1E,F show the TEM micrographs of uncoated RXS-SKNPs and GT-RXS-SKNPs. RXS-SKNPs appeared as discrete, nearly spherical NPs with relatively uniform distribution. After GT coating, GT-RXS-SKNPs retained a generally spherical morphology but showed a larger apparent diameter. The PS observed by TEM was smaller than the DLS-derived hydrodynamic diameters, which is expected because TEM measures dried particle cores, whereas DLS reflects the hydrated particle diameter, including the solvation layer and surface-associated polymer chains.

### 3.2 Surface-accessible amino group assay

**FIGURE 2:**
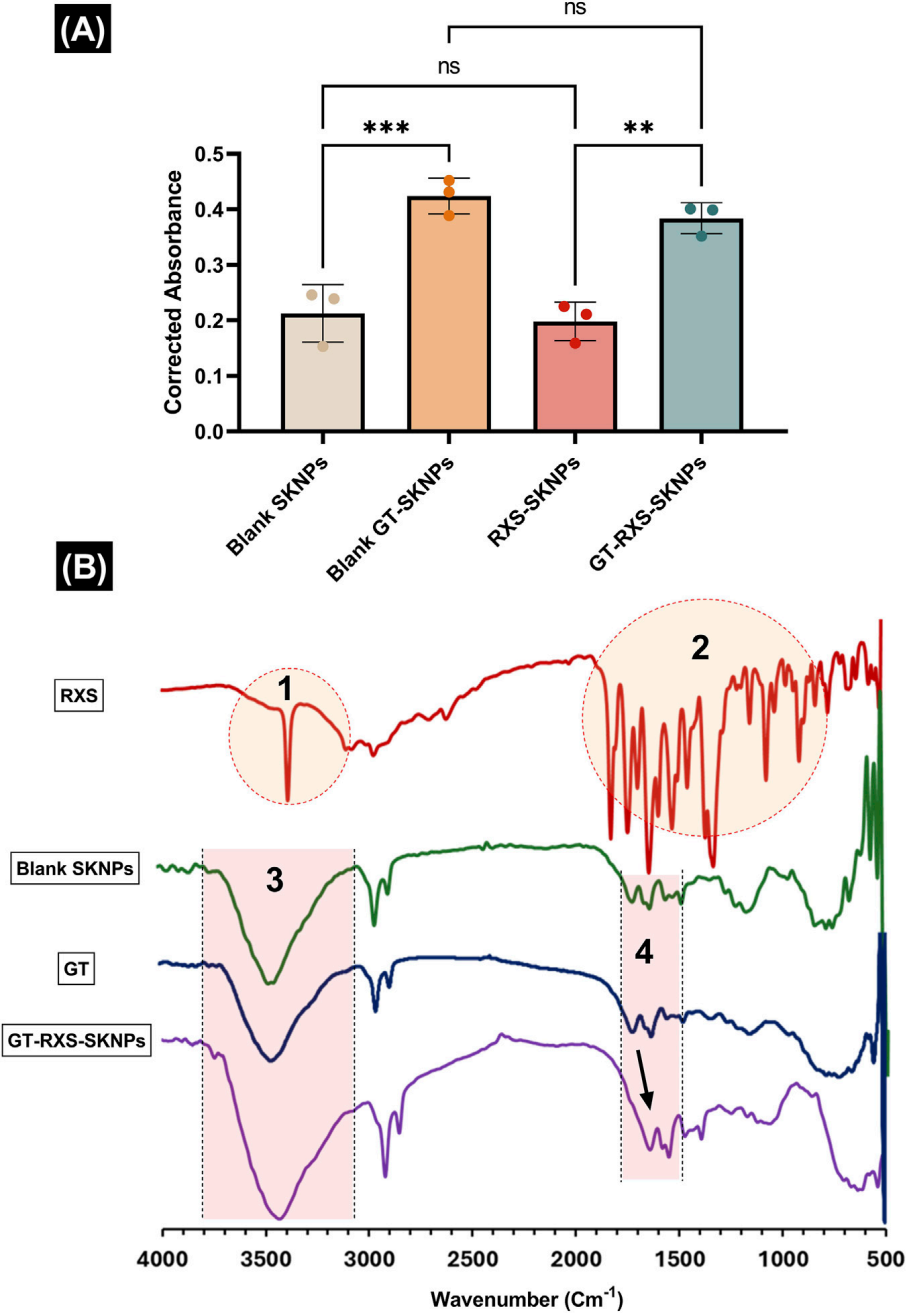
**(A)** Surface-accessible primary amino groups of RXS-SKNPs and GT-coated SKNPs determined using the TNBSA assay. Data are presented as mean corrected absorbance ±SD, n = 3. **p < 0.01, ***p < 0.001, ns: non-significant. **(B)** FTIR spectra of RXS, blank SKNPs, GT, GT-RXS-SKNPs, showing spectral changes consistent with RXS incorporation and gelatin–silk fibroin interactions.

The surface-accessible primary amino groups of RXS-SKNPs, GT-RXS-SKNPs, and their corresponding blank formulations were evaluated using the TNBSA assay (Figure 2A). Blank SKNPs showed a baseline corrected absorbance (*cA*) of 0.213 ± 0.052, which is expected because SK is a proteinaceous material and contains intrinsic amino groups. However, after GT coating, *cA* significantly increased (*p* < 0.001) to 0.424 ± 0.032 for blank GT-SKNPs, representing approximately a 1.99-fold increase compared with uncoated blank SKNPs. A similar pattern was observed after RXS loading. GT-RXS-SKNPs exhibited a significantly higher (*p* < 0.01) *cA* of 0.384 ± 0.028. These results indicate an increase in the accessible surface amino signal after GT coating, confirming the formation of the GT layer around SKNPs. Moreover, there was no significant difference in the *cA* between blank formulations and RXS-loaded formulations, indicating that RXS loading neither alters the accessible surface amino signal nor prevents the formation of a gelatin-enriched surface layer. As a result, the observed changes are primarily attributable to the presence of the GT coating.

### 3.3 FTIR

Figure 2B shows the FTIR spectra of raw RXS, blank SKNPs, GT, and GT-RXS-SKNPs. RXS showed characteristic bands at 3,351 cm^-1^ corresponding to the N-H stretch and several intense bands in the fingerprint region between approximately 1700 and 1,000 cm^-1^. These bands are mainly related to carbonyl stretching, aromatic skeletal vibrations, C–N stretching, and C–O/C–O–C vibrations of RXS (Saber et al., 2025).

Blank SKNPs spectrum showed a broad band at 3,300–3,500 cm^-1^, corresponding to overlapping O–H and N–H stretching vibrations. Additional characteristic amide bands were observed in the regions of approximately 1,610 cm^-1^, 1,520 cm^-1^, and 1,200 cm^-1^, corresponding to amide I, amide II, and amide III vibrations, respectively. The FTIR spectrum of GT also showed a broad O–H/N–H stretching band around 3,300–3,500 cm^-1^, together with characteristic protein amide bands. A broad amide III/fingerprint region was also detected around 1,200–1,300 cm^-1^. These findings confirm the characteristic collagen-derived protein structure of gelatin.

In the spectrum of GT-RXS-SKNPs, four main spectral differences were observed. Two of these differences were identified relative to raw RXS. First, the characteristic N–H stretching band of raw RXS at 3,351 cm^-1^ disappeared in the spectrum of GT-RXS-SKNPs (label 1, Figure 2B). Second, the characteristic RXS bands in the fingerprint region (1,000–1700 cm^-1^) were absent or markedly attenuated in the final formulation (label 2, Figure 2B). These two observations suggest that RXS was successfully incorporated into the SKNP matrix and was no longer present in its free crystalline form, indicating molecular dispersion and/or amorphization within the nanoparticle system.

The other two spectral differences were associated with the protein-based components, silk fibroin and gelatin. The broad band at 3,300–3,500 cm^-1^, corresponding to overlapping O–H/N–H stretching vibrations in blank SKNPs and GT, became broader in the spectrum of GT-RXS-SKNPs (label 3, Figure 2B), suggesting the contribution of gelatin and the possible formation of hydrogen bonding between GT and SK. In addition, the amide I and amide II bands in the 1,500–1700 cm^-1^ region were retained in the GT-RXS-SKNP spectrum but showed broadening and slight shifting (label 4, Figure 2B), supporting intermolecular interactions between the gelatin shell and the silk fibroin nanoparticle core. Thus, these FTIR changes confirm both successful gelatin coating and successful incorporation of RXS within the SKNPs matrix.

### 3.4 MMP-2-triggered colloidal remodeling study

**FIGURE 3:**
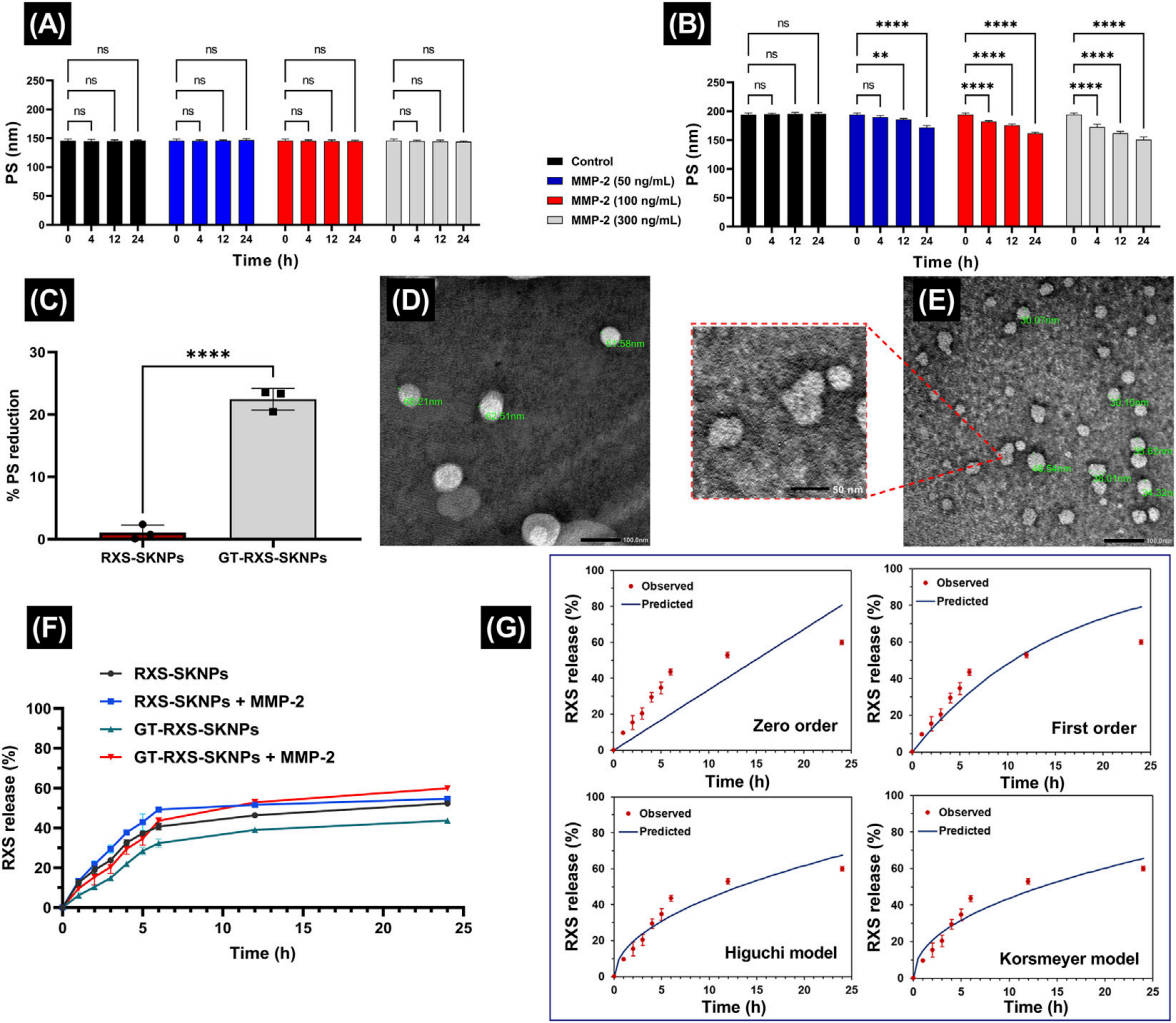
MMP-2-triggered colloidal remodeling and release behavior of RXS-SKNPs and GT-RXS-SKNPs. **(A,B)** Time-dependent hydrodynamic PS changes of RXS-SKNPs and GT-RXS-SKNPs after incubation with 50, 100, and 300 ng/mL activated MMP-2 for 24 h. **(C)** PS reduction (%) after 24 h incubation with 300 ng/mL MMP-2, calculated relative to the initial size of each formulation. **(D,E)** TEM images of RXS-SKNPs and GT-RXS-SKNPs after 24 h MMP-2 incubation. The inset in panel E shows a magnified region of GT-RXS-SKNPs. **(F)** *In vitro* RXS release profiles from uncoated and GT-coated nanoparticles in the absence and presence of MMP-2. **(G)** Release kinetic fitting of GT-RXS-SKNPs in MMP-2-containing medium. Data are presented as mean ± SD, n = 3. Ns: non-significant; **p < 0.01; ****p < 0.0001.

The MMP-2-responsive behavior of the GT coat was investigated by exposing both GT-coated and uncoated RXS-SKNPs to varying concentrations of MMP-2 (50, 100, and 300 ng/mL), and changes in PS were measured over 24 h (Figure 3). These concentrations were selected as previous studies reported comparable MMP-2 concentrations within the PF microenvironment (Bormann et al., 2022; Inoue et al., 2023). As shown in Figure 3A, the uncoated RXS-SKNPs showed non-significant differences in PS over 24 h. After 24 h of exposure to the highest MMP-2 concentration (300 ng/mL), the PS of RXS-SKNPs did not significantly decrease from 145.73 ± 2.75 nm to 144.13 ± 0.99 nm. These results indicate the colloidal stability of SKNPs after MMP-2 exposure.

In contrast, the GT-RXS-SKNPs exhibited a significant change in PS at all selected MMP-2 concentrations, with the exception of 4 h exposure to 50 ng/mL MMP-2 (Figure 3B). After 24 h of exposure to 300 ng/mL MMP-2, the PS of GT-RXS-SKNPs significantly decreased from 194.26 ± 2.94 nm to 150.66 ± 4.66 nm. These results highlight the MMP-2-responsive behavior of the GT coat. MMP-2, also known as gelatinase A, is capable of cleaving gelatin and several collagen-related matrix substrates (Xu Y. et al., 2019), which provides a mechanistic basis for the observed reduction in the PS after enzyme exposure.

To further clarify the comparative MMP-2 responsiveness of the two formulations, the size response after 24 h exposure to 300 ng/mL MMP-2 was normalized to the initial PS of each formulation. As shown in Figure 3C, uncoated RXS-SKNPs showed only minimal PS reduction after MMP-2 exposure, whereas GT-RXS-SKNPs exhibited a significantly greater percentage reduction in hydrodynamic PS. This finding supports that the observed MMP-2-triggered size evolution was mainly associated with enzymatic remodeling of the gelatin shell rather than nonspecific instability of the SKNPs core.

TEM analysis after 24 h incubation with activated MMP-2 further supported this interpretation. Uncoated RXS-SKNPs maintained their particulate morphology with no evident enzyme-associated structural disruption (Figure 3D), whereas GT-RXS-SKNPs showed apparent surface remodeling and reduced particle dimensions after MMP-2 exposure (Figure 3E). These morphological observations are consistent with partial gelatin-shell degradation/relaxation under gelatinase-rich conditions. The zoomed region shows irregular, partially fragmented particle morphology after 24 h MMP-2 exposure. This supports the idea that MMP-2 not only numerically reduced the DLS size but also caused visible surface disruption of the GT-coated NPs.

### 3.5 *In vitro* release study

The *in vitro* release study was conducted for RXS-SKNPs and GT-RXS-SKNPs under two different conditions: with or without MMP-2 exposure (Figure 3F). In an enzyme-free medium, although both formulations exhibited sustained RXS release for 24 h, the coated GT-RXS-SKNPs showed a significantly lower mean release percentage than the uncoated formulation. After 24 h, RXS-SKNPs released 52.40% ± 1.28% while GT-RXS-SKNPs showed a slower release pattern, reaching 43.70% ± 1.32%.

Regarding the release behavior of the uncoated formulation, the inherent structure of SK, characterized by alternating hydrophobic and hydrophilic domains, might cause slow release due to persistent β-sheet crystalline regions. A tight protein matrix with β-sheet-rich domains can reduce water penetration and impede drug diffusion from NPs (Hofmann et al., 2006). RXS’s physicochemical properties may also contribute to the obtained sustained release. RXS, a lipophilic acidic chemical with a great affinity for plasma proteins (Czock and Keller, 2022), may interact with proteinaceous matrices like SK via hydrophobic and hydrogen bonding. Interactions may reduce RXS mobility within the SK network, explaining the partial release observed within 24 h. For the coated formulation, the significantly lower release may be attributed to the presence of the GT coat, which can act as an additional diffusional barrier, limiting RXS release.

Upon exposure to MMP-2, the release behavior of RXS-SKNPs showed no significant difference compared to enzyme-free release behavior, reaching 54.69% ± 1.06% after 24 h, compared to 52.40% ± 1.28% within enzyme-free conditions. These results indicate that MMP-2 did not substantially alter the release behavior of the uncoated NPs. In contrast, GT-RXS-SKNPs showed a significant increase in the RXS release% (59.93% ± 1.30%) after 24 h compared to 43.70% ± 1.32% that was obtained under enzyme-free conditions. These results correlate with the MMP-2-triggered colloidal remodeling study, highlighting the proposed bioresponsive role of the GT coating.

After fitting the release data of GT-RXS-SKNPs + MMP-2 into different kinetic models (Figure 3G), the Higuchi order exhibited the highest *R* ^*2*^ (0.916 ± 0.02), followed by First order (0.803 ± 0.06), and Zero order (0.595 ± 0.005). These results suggest that diffusion contributed to the release of RXS. Furthermore, after fitting the release data to the Korsmeyer–Peppas model, the release exponent *n* was 0.505 ± 0.011, which is >0.45, indicating anomalous, non-Fickian transport (Nasr et al., 2023). This suggests that RXS release from GT-RXS-SKNPs in the presence of MMP-2 was governed by a combination of drug diffusion and matrix/shell relaxation or erosion.

### 3.6 Hemocompatibility and physical stability

**FIGURE 4:**
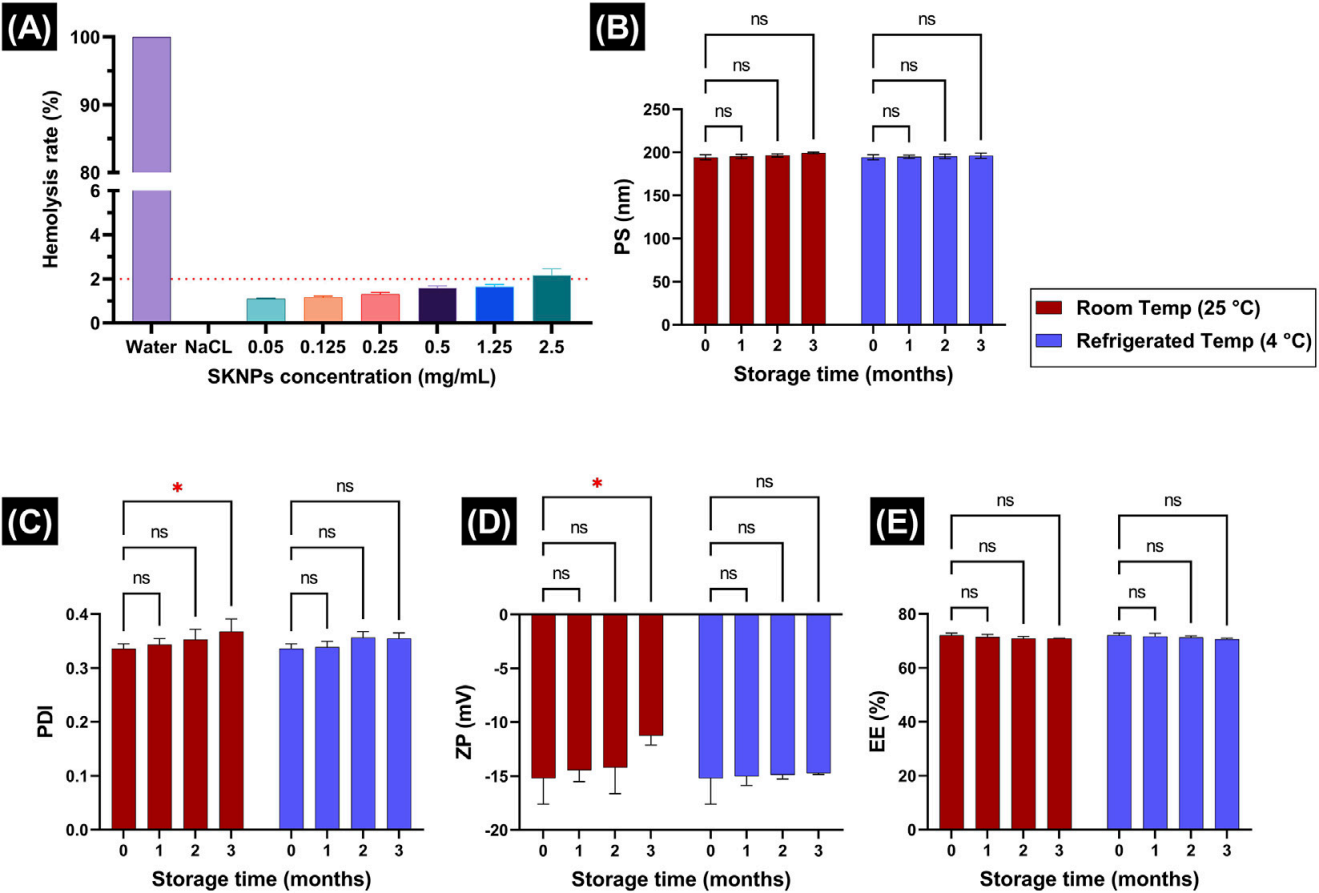
The mean hemolysis percentage of GT-RXS-SKNPs at concentrations range of 0.05–2.5 mg/mL, compared with distilled water as a positive control and normal saline as a negative control **(A)**. Storage stability of GT-RXS-SKNPs over 3 months at room temperature (25 °C) and refrigerated temperature (4 °C), expressed as changes in PS **(B)**, PDI **(C)**, ZP **(D)**, and EE% **(E)**. Data are presented as mean ± SD, n = 3. Ns: non-significant; *p < 0.05.

Figure 4A shows the hemocompatibility results for GT-RXS-SKNPs at concentrations ranging from 0.05 to 2.5 mg/mL. With the exception of a 2.5 mg/mL concentration, GT-RXS-SKNPs exhibited a hemolysis percentage <2% at all tested concentrations. The hemolysis percentage increased gradually in a concentration-dependent manner. At 0.05 mg/mL, the hemolysis % was 1.097% ± 0.028%, reaching 1.64% ± 0.102% at 1.25 mg/mL, indicating good hemocompatibility at these concentrations. At the highest tested concentration (2.5 mg/mL), hemolysis reached 2.17% ± 0.28%, indicating slight hemolysis; however, it remained far below the strongly hemolytic range (Gawlikowski et al., 2020). Therefore, GT-RXS-SKNPs can be considered non-hemolytic up to 1.25 mg/mL.

The physical stability of the optimized GT-RXS-SKNPs was evaluated for 3 months under two storage conditions: at 25 °C and 4 °C (Figures 4B–E). In both conditions, the formulations showed no significant differences in any of the tested parameters, except for ZP and PDI after 3 months of storage at room temperature. PDI increased significantly, and ZP decreased significantly compared to their initial value. These results indicate that the formulation maintained its nanoscale size, negative surface charge, and drug entrapment capacity over 3 months. Although slight changes in PDI and zeta potential were observed at room temperature, refrigerated storage provided better preservation of colloidal properties. Therefore, storage at 4 °C may be more suitable for maintaining the long-term physical stability of GT-RXS-SKNPs.

### 3.7 In vivo

#### 3.7.1 *In vivo* lung distribution study

**FIGURE 5:**
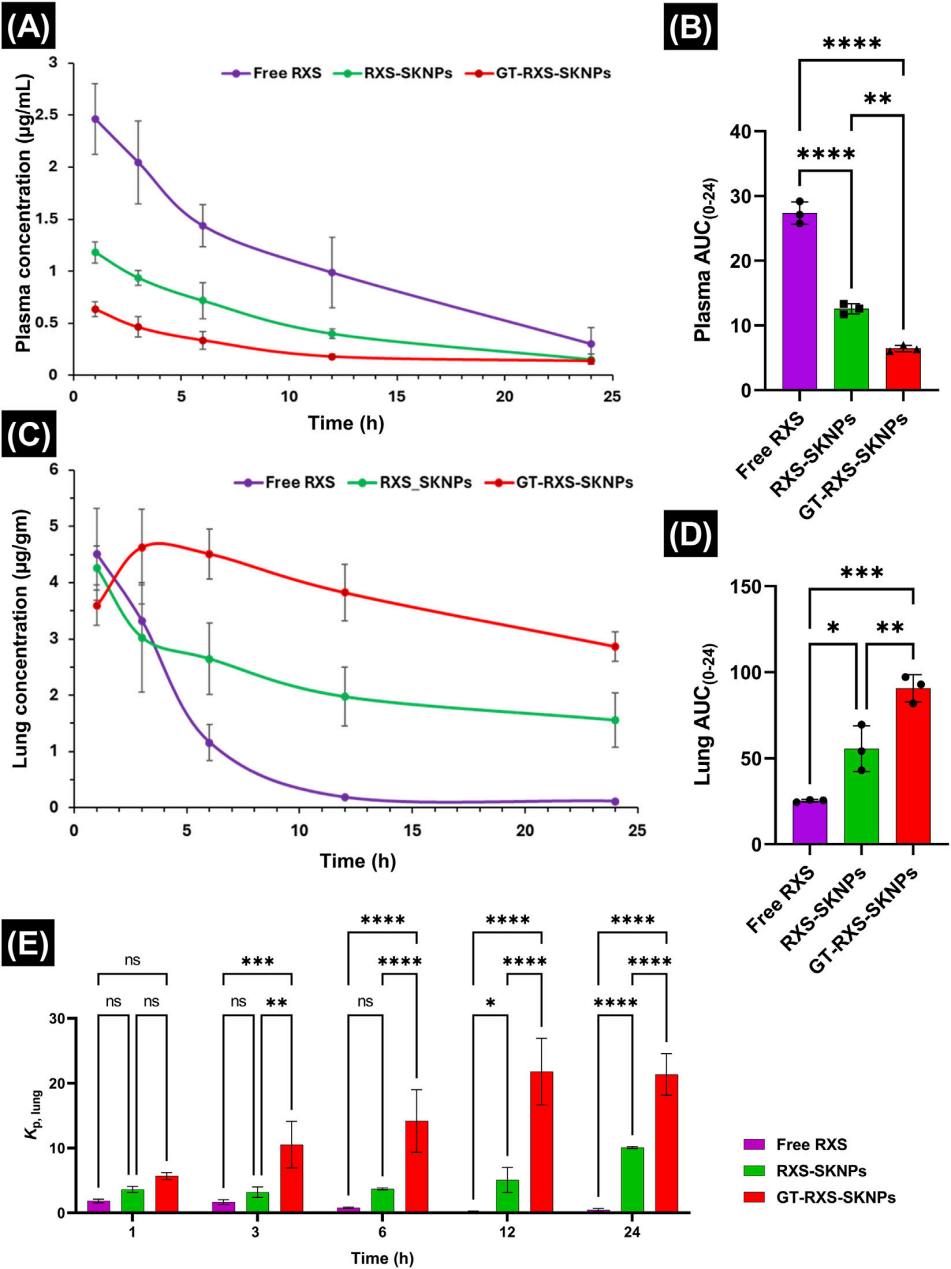
*In vivo* plasma pharmacokinetics and lung distribution of RXS after single IV administration of free RXS, uncoated RXS-SKNPs, and GT-RXS-SKNPs at an equivalent RXS dose of 5 mg/kg to PF-diseased male Wistar rats. **(A)** Plasma concentration–time profiles. **(B)** Plasma AUC_0_–_24_. **(C)** Lung concentration–time profiles. **(D)** Lung AUC_0_–_24_. **(E)** Apparent lung-to-plasma partition index, *Kp*, calculated as lung concentration/plasma concentration at each time point. Data are presented as mean ± SD, n = 3. Ns: non-significant; *p < 0.05; **p < 0.01; ***p < 0.001; ****p < 0.0001.

The distribution of RXS within lung tissue was investigated by comparing plasma and lung concentration-time profiles, calculating plasma and lung *AUC*_*0-24*_, and determining lung partition coefficients (*Kp*) at different time points after a single IV administration of free RXS, RXS-SKNPs, and GT-RXS-SKNPs, all at an RXS dose equivalent to 5 mg/kg (Figure 5). As shown in Figure 5A, free RXS exhibited the highest plasma concentration, 2.46 ± 0.34 μg/mL, at 1 h. At the same time point, RXS-SKNPs and GT-RXS-SKNPs gave plasma concentrations of 1.18 ± 0.10 μg/mL and 0.64 ± 0.07 μg/mL, respectively. Furthermore, the plasma AUC_0-24_ for free RXS was significantly higher (27.36 ± 1.71 μg h/mL) than for RXS-SKNPs (12.56 ± 0.79 μg h/mL) and GT-RXS-SKNPs (6.44 ± 0.49 μg h/mL, Figure 5B). The relative systemic exposure (*RSe*) of RXS-SKNPs and GT-RXS-SKNPs to free RXS was 0.458 ± 0.006 and 0.235 ± 0.003, respectively. Compared with the other nanoformulation, the GT-coated formula exhibited an *RSe* value of 0.512 ± 0.015. These results indicate that incorporating RXS into NPs, particularly after GT coating, significantly decreased circulating RXS exposure and may reduce systemic side effects associated with non-specific HIF-1 activation.

Regarding the lung concentration-time profile (Figure 5C), at the first time point, the free RXS and RXS-SKNPs showed a significantly higher lung concentration of 4.50 ± 0.82 μg/g and 4.26 ± 0.39 μg/g, respectively, compared to GT-RXS-SKNPs (3.60 ± 0.36 μg/g). However, GT-RXS-SKNPs exhibited the highest lung concentration at 3 h (4.63 ± 0.67 μg/g) and maintained a significantly higher lung concentration, reaching 2.87 ± 0.26 μg/g at 24 h, compared to 1.56 ± 0.48 μg/g for uncoated SKNPs and 0.11 ± 0.01 μg/g for free RXS. The lung AUC_0-24_ values highlight the superior pulmonary exposure of GT-RXS-SKNPs, which enhances drug concentration within pulmonary tissues (Figure 5D). Free RXS showed the lowest lung AUC_0-24_ of 25.28 ± 0.77 μg h/g. Uncoated RXS-SKNPs significantly increased lung AUC_0-24_ to 55.57 ± 13.31 μg h/g, corresponding to 2.2-fold compared with free RXS. GT-RXS-SKNPs produced the highest lung AUC_0-24_ of 90.72 ± 7.86 μg h/g, corresponding to a 3.58-fold enhancement compared with free RXS and approximately a 1.71-fold enhancement compared with uncoated RXS-SKNPs.

The lung kinetic parameters further supported the sustained pulmonary residence of the nanoformulations. Free RXS exhibited a short apparent lung *t*_*1/2*_ of 4.17 ± 0.06 h and *MRT* of 4.62 ± 0.27 h. Uncoated RXS-SKNPs markedly prolonged lung residence, with an apparent lung *t*_*1/2*_ of 23.85 ± 3.68 h and *MRT* of 33.51 ± 5.39 h. GT-RXS-SKNPs showed the longest lung persistence, with an apparent lung *t*_*1/2*_ of 28.85 ± 5.23 h and *MRT* of 42.13 ± 8.34 h. Thus, GT coating increased the apparent lung *MRT* approximately 9.1-fold compared with free RXS and approximately 1.26-fold compared with uncoated RXS-SKNPs. Therefore, GT coating improved both the localization and duration of pulmonary exposure, with the strongest effect being prolongation of lung residence rather than only elevation of peak concentration.

A particularly important finding was the opposite behavior of GT-RXS-SKNPs in plasma and lung. Although GT-RXS-SKNPs showed the lowest plasma AUC_0_–_24_, they produced the highest lung AUC_0_–_24_. This indicates that the coated formulation did not simply increase total systemic exposure; instead, it shifted RXS disposition toward the lung compartment. This was further confirmed by the drug distribution index values. GT-RXS-SKNPs exhibited a *DDI* value of 4.85 ± 1.31 and 3.36 ± 1.07, compared to free RXS and uncoated RXS-SKNPs, respectively. These results indicate that the coated formulation maintained lung exposure while plasma concentrations continued to decline. This was further confirmed by the time-dependent lung-to-plasma partition index (Kp), which provided additional evidence of preferential lung retention (Figure 5E). Free RXS showed low and declining Kp values over time, decreasing from 1.83 ± 0.29 at 1 h to 0.45 ± 0.23 at 24 h. Uncoated RXS-SKNPs produced higher Kp values, increasing from 3.61 ± 0.47 at 1 h to 10.09 ± 0.15 at 24 h. GT-RXS-SKNPs showed the highest Kp values at all time points, increasing from 5.68 ± 0.55 at 1 h to 21.35 ± 3.22 at 24 h.

#### 3.7.2 GT-RXS-SKNPs attenuated bleomycin-induced histopathological lung injury, collagen deposition, and myofibroblast activation

**FIGURE 6:**
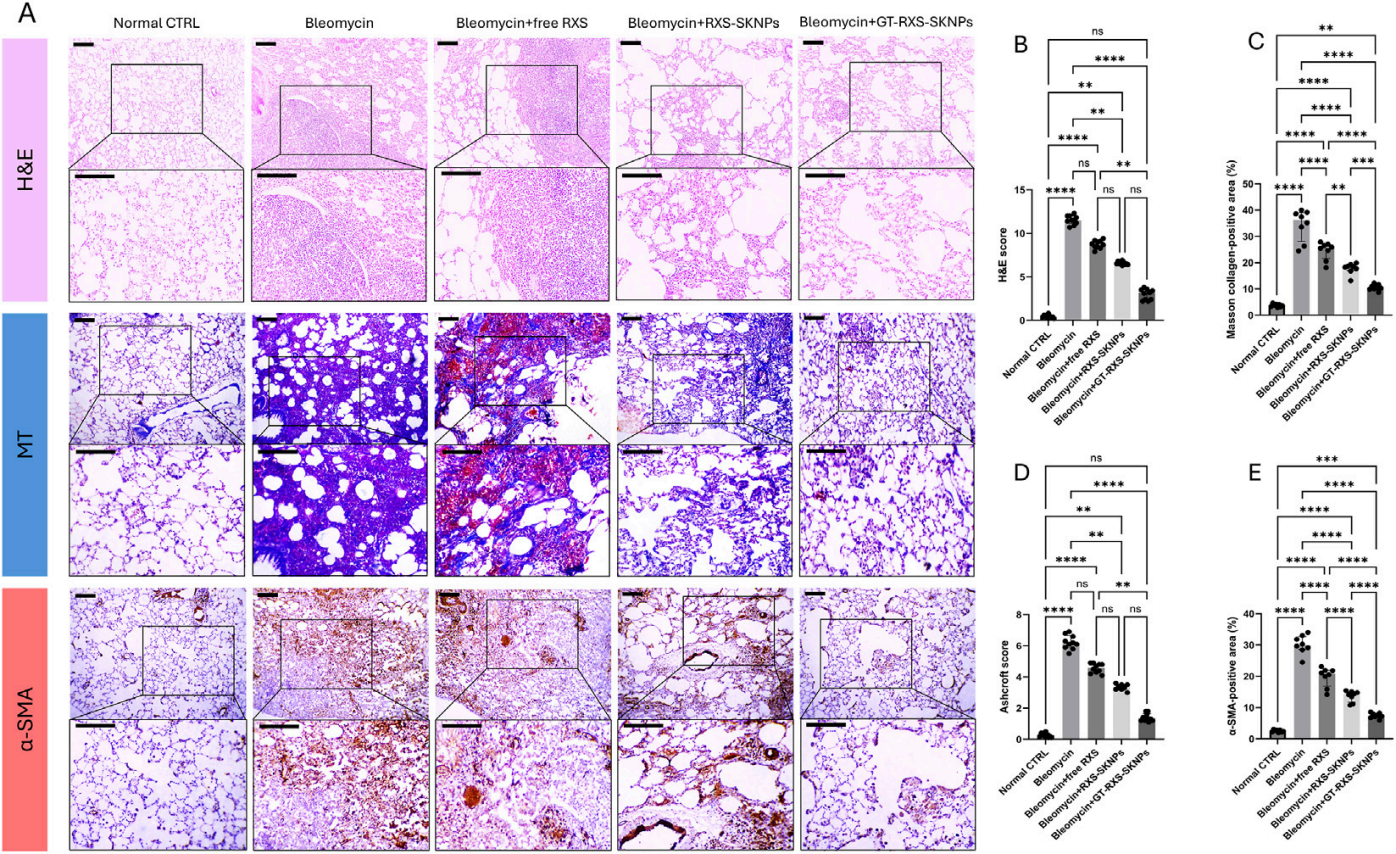
Histopathological and immunohistochemical assessment of lung fibrosis. **(A)** Representative H&E−, Masson trichrome (MT)-, and α-SMA-stained lung sections from normal control and bleomycin-challenged rats treated with free RXS, RXS-SKNPs, or GT-RXS-SKNPs at an equivalent RXS dose of 5 mg/kg. Boxed areas are shown at higher magnification. Scale bar = 100 μm. **(B–E)** Quantification of H&E injury score, Masson collagen-positive area, Ashcroft fibrosis score, and α-SMA-positive area, respectively. Data are shown as individual animal values with group summaries (n = 8 rats/group). H&E and Ashcroft scores were analyzed by Kruskal–Wallis/Dunn’s test; Masson and α-SMA areas were analyzed by one-way ANOVA/Tukey’s test. ns, non-significant; **p < 0.01; ***p < 0.001; ****p < 0.0001.

Representative H&E-stained sections showed preserved alveolar architecture, thin alveolar septa, and minimal inflammatory-cell infiltration in the normal control group (Figure 6A; H&E panel). In contrast, the bleomycin group showed severe histopathological injury characterized by inflammatory-cell infiltration, septal thickening, alveolar collapse, consolidation, and marked distortion of normal lung architecture. Free RXS partially improved the histological appearance, whereas RXS-SKNPs produced a greater preservation of alveolar structure. The most evident protection was observed in the GT-RXS-SKNPs group, in which the alveolar architecture was largely restored and inflammatory/fibrotic distortion was markedly reduced.

Semi-quantitative H&E scoring confirmed these histological observations (Figure 6B). Bleomycin significantly increased the H&E injury score compared with the normal control group. Free RXS reduced the H&E score compared with the bleomycin group (although non significant), and further reductions were observed in the RXS-SKNPs and GT-RXS-SKNPs groups. Although GT-RXS-SKNPs produced the lowest numerical H&E injury score among the bleomycin-challenged treatment groups, the Kruskal–Wallis test followed by Dunn’s multiple-comparison correction did not fully discriminate between the uncoated RXS-SKNPs and GT-RXS-SKNPs groups. Although the multiplicity-adjusted Dunn’s *post hoc* analysis did not fully discriminate between the uncoated RXS-SKNPs and GT-RXS-SKNPs groups for the H&E score, GT-RXS-SKNPs showed the lowest numerical histopathological score and the strongest overall improvement when interpreted together with the quantitative morphometric and biochemical endpoints.

Masson trichrome staining showed minimal collagen deposition in the normal control lung, whereas bleomycin produced extensive collagen accumulation throughout the injured parenchyma (Figure 6A; MT panel). Quantitative image analysis demonstrated a marked increase in Masson collagen-positive area in the bleomycin group compared with the normal control group (Figure 6C). Free RXS significantly reduced collagen-positive area, while RXS-SKNPs produced a stronger reduction. GT-RXS-SKNPs produced the greatest suppression of collagen deposition and significantly reduced Masson collagen-positive area compared with uncoated RXS-SKNPs, indicating that gelatin coating enhanced the antifibrotic effect of the RXS-loaded nanocarrier.

The modified Ashcroft score further supported the development of established pulmonary fibrosis after bleomycin administration and its attenuation by RXS-based treatment (Figure 6D). Bleomycin markedly increased the Ashcroft score compared with normal control rats, confirming the establishment of pulmonary fibrosis. Free RXS produced only a numerical reduction in the Ashcroft score compared with the bleomycin group, but this effect did not reach statistical significance. In contrast, RXS-SKNPs significantly reduced the Ashcroft score compared with bleomycin, while GT-RXS-SKNPs produced the lowest numerical Ashcroft score among the bleomycin-challenged treatment groups. However, Kruskal–Wallis analysis followed by Dunn’s multiple-comparison correction did not show a significant difference between the uncoated RXS-SKNPs and GT-RXS-SKNPs groups for the Ashcroft score, consistent with the limited sensitivity of ordinal fibrosis scoring for adjacent treatment effects. Nevertheless, GT-RXS-SKNPs showed the lowest numerical Ashcroft score and the strongest overall improvement when interpreted together with the quantitative morphometric and biochemical endpoints.

α-SMA immunohistochemistry revealed marked myofibroblast accumulation in the bleomycin group, as shown by intense brown DAB-positive staining in fibrotic/interstitial areas (Figure 6A; α-SMA panel). Quantitative analysis showed that bleomycin significantly increased α-SMA-positive area compared with normal control rats (Figure 6E). Free RXS reduced α-SMA immunostaining, and RXS-SKNPs produced a stronger decrease. GT-RXS-SKNPs produced the greatest reduction in α-SMA-positive area and significantly reduced α-SMA staining compared with uncoated RXS-SKNPs, indicating superior suppression of myofibroblast activation.

#### 3.7.3 GT-RXS-SKNPs suppressed BALF inflammatory cytokines

**FIGURE 7:**
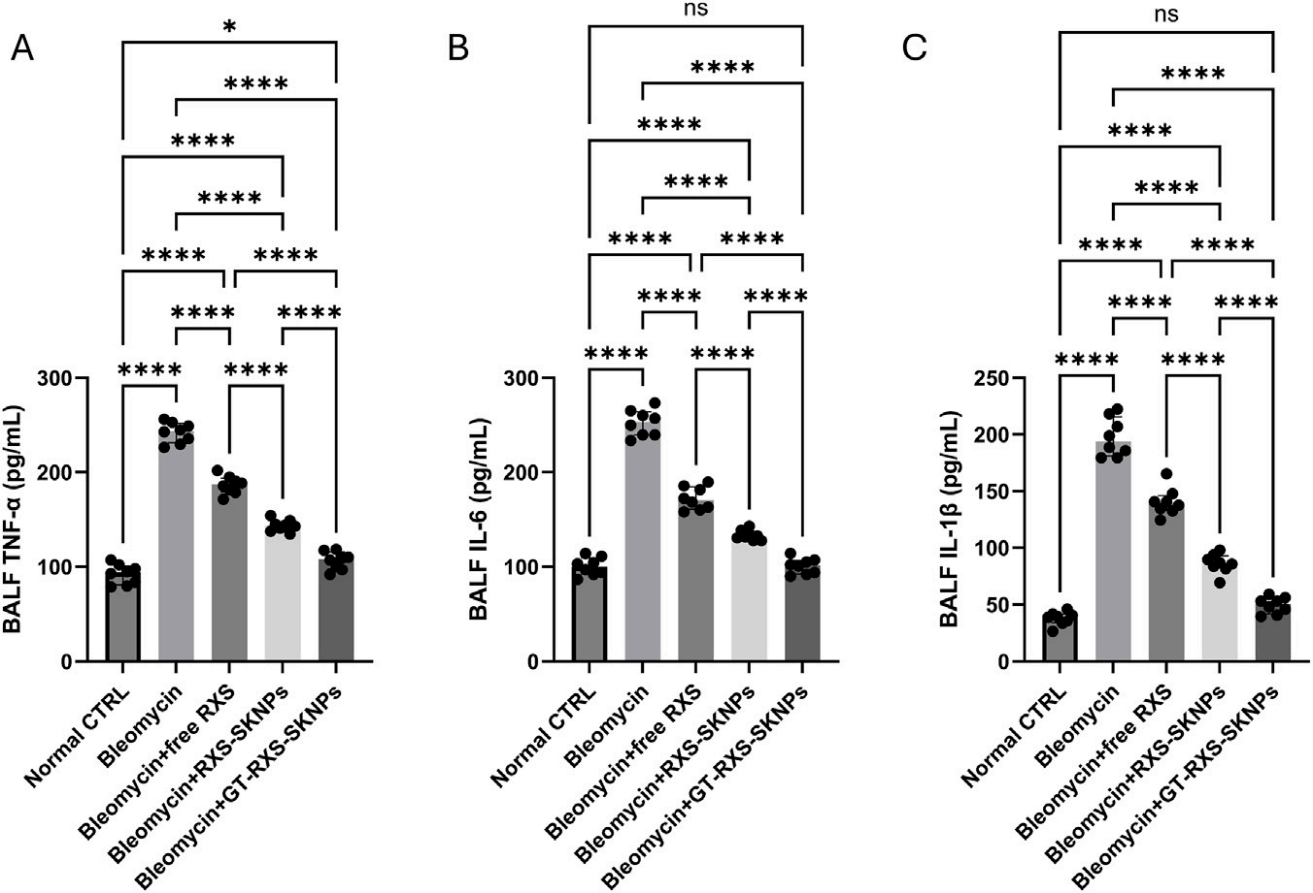
BALF inflammatory cytokine levels in normal control rats and bleomycin-challenged rats treated with free RXS, uncoated RXS-SKNPs, or GT-RXS-SKNPs at an equivalent RXS dose of 5 mg/kg. BALF was collected on day 28 after bleomycin instillation, and cytokine concentrations were measured by ELISA. **(A)** BALF TNF-α concentration. **(B)** BALF IL-6 concentration. **(C)** BALF IL-1β concentration. Data are presented as mean ± SD, n = 8 rats/group. Data were analyzed using one-way ANOVA followed by Tukey’s multiple-comparison test. Statistical comparisons are shown by brackets. ns: non-significant; *p < 0.05; **p < 0.01; ***p < 0.001; ****p < 0.0001.

Bleomycin challenge significantly increased BALF TNF-α, IL-6, and IL-1β concentrations compared with the normal control group, confirming persistent inflammatory activation at the fibrotic endpoint (Figures 7A–C). Free RXS significantly reduced all three BALF cytokines compared with the bleomycin group. The anti-inflammatory effect was stronger after incorporation of RXS into SKNPs, as shown by lower BALF TNF-α, IL-6, and IL-1β levels in the RXS-SKNPs group compared with free RXS.

GT-RXS-SKNPs produced the strongest suppression of BALF inflammatory cytokines among the treated groups. In all three cytokine panels, GT-RXS-SKNPs significantly reduced BALF cytokine levels compared with uncoated RXS-SKNPs. These findings indicate that gelatin-coated RXS-SKNPs not only attenuated structural fibrotic remodeling but also more effectively limited residual inflammatory signaling in the bronchoalveolar compartment.

#### 3.7.4 GT-RXS-SKNPs reduced lung hydroxyproline, TGF-β1, and gelatinase-related remodeling markers

**FIGURE 8:**
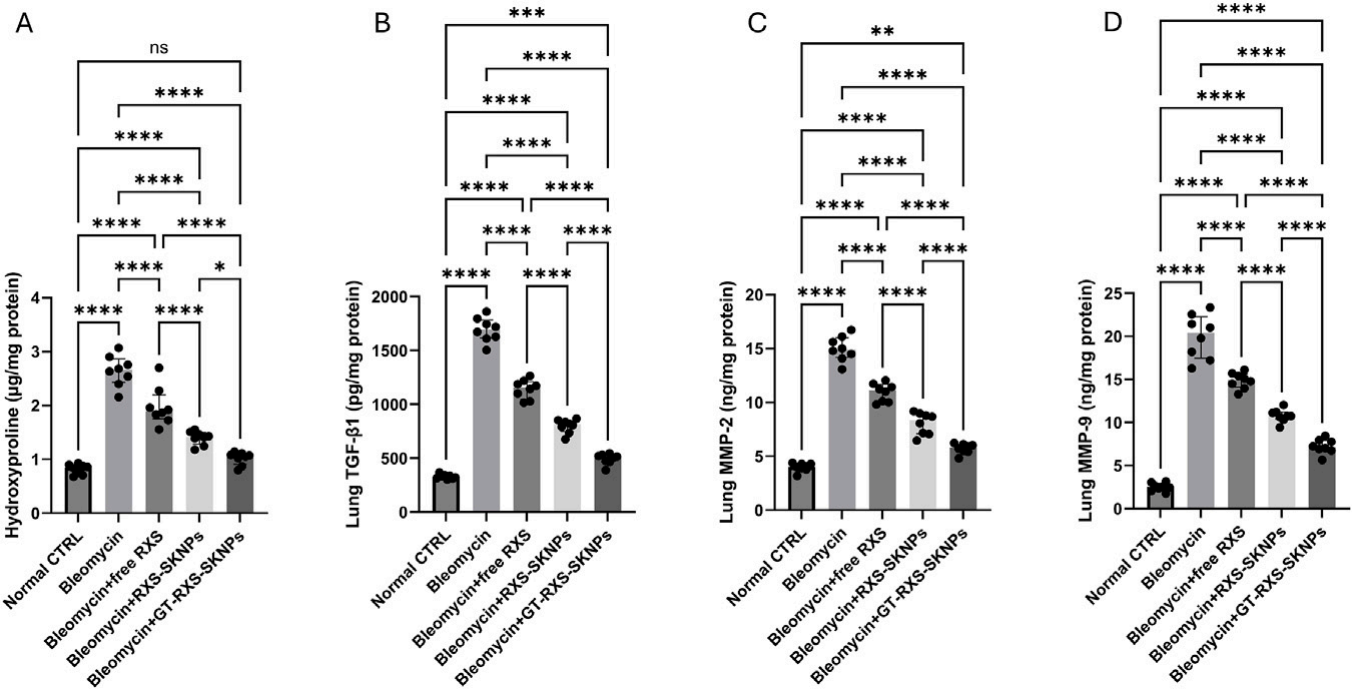
Lung collagen burden, profibrotic signaling, and gelatinase-related remodeling markers in normal control rats and bleomycin-challenged rats treated with free RXS, uncoated RXS-SKNPs, or GT-RXS-SKNPs at an equivalent RXS dose of 5 mg/kg. Lung tissues were collected on day 28 after bleomycin instillation. **(A)** Lung hydroxyproline content. **(B)** Lung TGF-β1 concentration. **(C)** Lung MMP-2 concentration. **(D)** Lung MMP-9 concentration. Data are presented as mean ± SD, n = 8 rats/group. Data were analyzed using one-way ANOVA followed by Tukey’s multiple-comparison test. Statistical comparisons are shown by brackets. ns: non-significant; *p < 0.05; **p < 0.01; ***p < 0.001; ****p < 0.0001.

Lung hydroxyproline content was significantly increased in the bleomycin group compared with the normal control group, confirming increased collagen burden in fibrotic lung tissue (Figure 8A). Free RXS significantly decreased hydroxyproline content compared with the bleomycin group. RXS-SKNPs produced a further reduction, and GT-RXS-SKNPs showed the lowest hydroxyproline values among the bleomycin-challenged treatment groups. The gelatin-coated formulation significantly reduced hydroxyproline content compared with uncoated RXS-SKNPs, supporting the superiority of GT-RXS-SKNPs in limiting collagen accumulation.

Lung TGF-β1 levels were markedly elevated after bleomycin administration, consistent with activation of a profibrotic tissue microenvironment (Figure 8B). Free RXS significantly reduced TGF-β1 levels compared with the bleomycin group. RXS-SKNPs produced a stronger reduction than free RXS, while GT-RXS-SKNPs produced the greatest decrease and significantly reduced lung TGF-β1 compared with uncoated RXS-SKNPs. These findings indicate that improved pulmonary delivery of RXS was associated with stronger suppression of TGF-β1-linked profibrotic remodeling.

Bleomycin significantly increased lung total MMP-2 and total MMP-9 levels compared with normal control rats, supporting the presence of a gelatinase-enriched remodeling microenvironment in the fibrotic lung (Figures 8C,D). Free RXS and RXS-SKNPs reduced both gelatinase markers compared with bleomycin. GT-RXS-SKNPs produced the lowest MMP-2 and MMP-9 levels among the treated groups and significantly decreased each marker compared with uncoated RXS-SKNPs, supporting the superior ability of the gelatin-coated formulation to attenuate matrix-remodeling responses in bleomycin-induced pulmonary fibrosis.

#### 3.7.5 GT-RXS-SKNPs reduced lung MDA and restored GSH and SOD antioxidant responses

**FIGURE 9:**
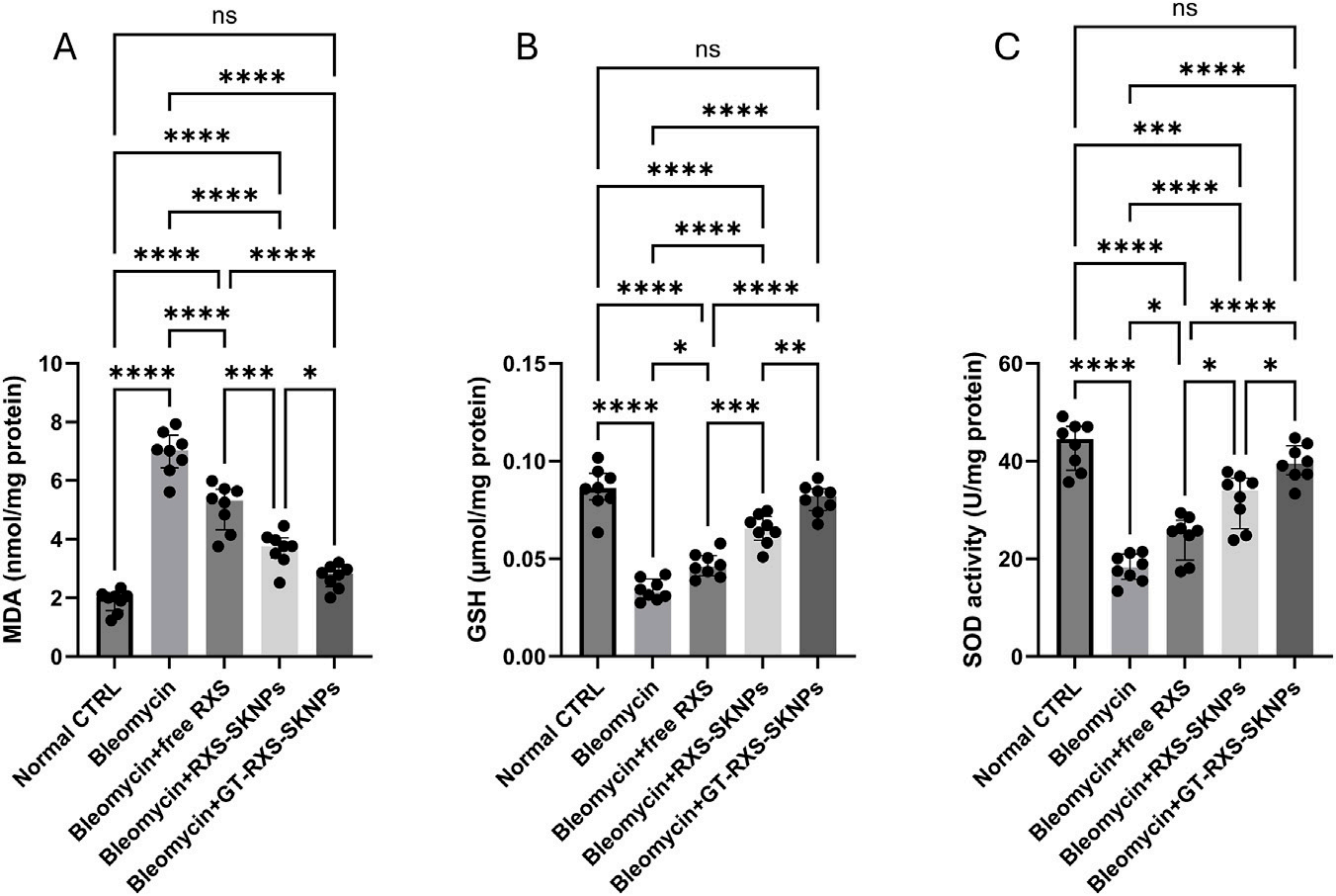
Lung oxidative-stress and antioxidant-defense markers in normal control rats and bleomycin-challenged rats treated with free RXS, uncoated RXS-SKNPs, or GT-RXS-SKNPs at an equivalent RXS dose of 5 mg/kg. Lung tissues were collected on day 28 after bleomycin instillation. **(A)** Lung MDA content **(B)** Lung GSH levels. **(C)** Lung SOD activity. Data are presented as mean ± SD, n = 8 rats/group. Data were analyzed using one-way ANOVA followed by Tukey’s multiple-comparison test. Statistical comparisons are shown by brackets. ns: non-significant; *p < 0.05; **p < 0.01; ***p < 0.001; ****p < 0.0001.

The effect of the different RXS formulations on oxidative stress in lung tissue was evaluated by measuring MDA content, GSH level, and SOD activity (Figures 9A–C). Bleomycin challenge markedly disrupted the pulmonary oxidant/antioxidant balance, as shown by a significant increase in lung MDA content and significant depletion of both GSH and SOD activity compared with the normal control group. As shown in Figure 9A, bleomycin significantly increased MDA levels, confirming enhanced lipid peroxidation in fibrotic lung tissue. Free RXS significantly reduced MDA compared with the bleomycin group, while RXS-SKNPs produced a stronger reduction. GT-RXS-SKNPs showed the greatest suppressive effect on MDA and significantly reduced MDA compared with both free RXS and uncoated RXS-SKNPs. Similarly, bleomycin significantly decreased lung GSH levels compared with normal control rats (Figure 9B). Free RXS partially restored GSH, whereas RXS-SKNPs produced a more pronounced increase. GT-RXS-SKNPs produced the highest GSH level among the bleomycin-challenged treatment groups and significantly increased GSH compared with uncoated RXS-SKNPs. Consistent with these findings, bleomycin significantly reduced lung SOD activity compared with the normal control group (Figure 9C). Free RXS significantly increased SOD activity relative to bleomycin, while RXS-SKNPs produced a greater improvement. GT-RXS-SKNPs showed the strongest restoration of SOD activity and significantly increased SOD compared with uncoated RXS-SKNPs. Collectively, these findings demonstrate that gelatin-coated RXS-SKNPs most effectively attenuated bleomycin-induced oxidative stress by reducing lipid peroxidation and restoring antioxidant defense mechanisms in lung tissue.

#### 3.7.6 GT-RXS-SKNPs produced the highest integrated fibrosis recovery across histological, inflammatory, remodeling, and oxidative-stress endpoints

To further compare the global therapeutic performance of the different RXS formulations, percent fibrosis recovery was calculated for all measured efficacy endpoints using the normal control and bleomycin groups as the reference limits. For endpoints increased by bleomycin, including Ashcroft score, H&E score, Masson collagen-positive area, α-SMA-positive area, BALF cytokines, lung hydroxyproline, TGF-β1, MMP-2, MMP-9, and MDA, recovery was calculated as the percentage reduction of the bleomycin-induced increase toward the normal-control value. For endpoints reduced by bleomycin, namely, GSH and SOD, recovery was calculated as the percentage restoration of the bleomycin-induced depletion toward the normal-control value.

Across the structural fibrosis endpoints, GT-RXS-SKNPs produced the highest recovery in Ashcroft score, H&E score, Masson collagen-positive area, α-SMA-positive area, and hydroxyproline content. The mean integrated recovery across these histological, collagen, and myofibroblast-related endpoints was 81.3% for GT-RXS-SKNPs, compared with 55.0% for uncoated RXS-SKNPs and 31.7% for free RXS. A similar pattern was observed for inflammatory markers, where GT-RXS-SKNPs achieved 99.7%, 92.7%, and 90.5% recovery for BALF IL-6, IL-1β, and TNF-α, respectively. The mean inflammatory recovery was therefore 94.3% for GT-RXS-SKNPs, compared with 71.3% for RXS-SKNPs and 41.7% for free RXS.

For profibrotic and gelatinase-related remodeling markers, GT-RXS-SKNPs also produced the strongest recovery, reaching 87.9% for TGF-β1, 84.2% for MMP-2, and 73.0% for MMP-9. The mean recovery across these remodeling markers was 81.7% for GT-RXS-SKNPs, compared with 60.5% for RXS-SKNPs and 35.5% for free RXS. In addition, GT-RXS-SKNPs showed the highest oxidative-stress recovery, with 83.6% recovery for MDA, 90.5% for GSH, and 85.7% for SOD, giving a mean oxidative-stress recovery of 86.6%, compared with 60.2% for RXS-SKNPs and 29.0% for free RXS.

When all measured endpoints were integrated, GT-RXS-SKNPs achieved the highest overall fibrosis recovery index of 85.3%, followed by uncoated RXS-SKNPs at 60.8% and free RXS at 34.1% (Table 3). This integrated recovery analysis supports the conclusion that gelatin coating markedly enhanced the therapeutic performance of RXS-SKNPs, producing broad recovery across histopathological injury, collagen deposition, myofibroblast activation, inflammatory cytokine release, profibrotic signaling, gelatinase-associated remodeling, lipid peroxidation, and antioxidant defense.

**TABLE 3:** Percent fibrosis recovery across measured efficacy endpoints after treatment with free RXS, RXS-SKNPs, and GT-RXS-SKNPs.

Endpoint	Free RXS recovery (%)	RXS-SKNPs recovery (%)	GT-RXS-SKNPs recovery (%)
Ashcroft score	27.9	48.3	80.9
H&E score	25.2	44.6	77.0
Masson collagen-positive area	32.0	54.1	77.7
α-SMA-positive area	37.3	59.6	82.2
Lung hydroxyproline	36.3	68.2	88.9
BALF IL-6	52.6	78.3	99.7
BALF IL-1β	35.7	69.8	92.7
BALF TNF-α	37.0	65.8	90.5
Lung TGF-β1	40.4	65.6	87.9
Lung MMP-2	37.0	63.1	84.2
Lung MMP-9	29.3	52.7	73.0
Lung MDA	36.7	64.8	83.6
Lung GSH	24.8	59.9	90.5
Lung SOD	25.4	55.8	85.7
Overall mean recovery	34.1	60.8	85.3

For endpoints increased by bleomycin, percent recovery was calculated as: [(Bleomycin mean − Treatment mean)/(Bleomycin mean − Normal mean)] × 100. For GSH, and SOD, which were reduced by bleomycin, percent recovery was calculated as: [(Treatment mean − Bleomycin mean)/(Normal mean − Bleomycin mean)] × 100.

## 4 Discussion

The current study aimed to enhance the therapeutic effect of RXS for PF and to offer a new treatment option. To achieve this goal, RXS was incorporated into SKNPs and further coated with GT to produce a bioresponsive, injectable delivery system that improves pulmonary delivery while reducing systemic exposure. The selection of this delivery system was based on three complementary components. Firstly, SKNPs are biocompatible, protein-based carriers capable of incorporating drugs and sustaining release (Bao et al., 2026). Secondly, GT is an outer layer that can modulate surface properties and respond to gelatinase-rich pathological environments of PF (Kim et al., 2009; Inoue et al., 2023). Thirdly, the GT layer can be formed around SKNPs depending on electrostatic interactions between cationic GT and anionic SKNPs under mild conditions at room temperature, The *in vitro* MMP-2 studies provided direct evidence for the enzyme-responsive behavior of the GT-coated formulation. Uncoated RXS-SKNPs showed minimal PS changes after MMP-2 exposure, whereas GT-RXS-SKNPs showed a clear reduction in PS. In parallel, MMP-2 exposure increased RXS release from GT-RXS-SKNPs compared with enzyme-free conditions. These results indicate that the GT layer acted as a responsive outer shell that could be remodeled in the presence of MMP-2, allowing greater drug release under enzyme-rich conditions.

In the present study, the term “colloidal remodeling” refers to MMP-2-induced changes in the colloidal properties and surface architecture of GT-RXS-SKNPs rather than complete degradation of the SKNPs core. This remodeling was mainly reflected by the time- and concentration-dependent reduction in hydrodynamic PS, together with TEM-observed surface irregularity and reduced apparent particle dimensions after enzymatic exposure. Mechanistically, the GT shell acts as the MMP-2-sensitive component of the nanoplatform (Kim et al., 2009; Inoue et al., 2023). Upon exposure to activated MMP-2, partial cleavage and relaxation of the gelatin coating are expected to reduce the hydrated shell thickness and loosen the surface layer surrounding the SKNPs core. This explains the reduction in hydrodynamic size and the enhanced RXS release observed in MMP-2-containing medium. In contrast, uncoated RXS-SKNPs lack this gelatinase-sensitive outer layer and therefore showed minimal size reduction under the same enzymatic conditions. These findings support that MMP-2 responsiveness arises primarily from gelatin-shell remodeling rather than nonspecific instability of the silk fibroin nanoparticles.

The *in vivo* distribution data showed that GT-RXS-SKNPs reduced plasma exposure while increasing lung exposure compared with free RXS and uncoated RXS-SKNPs. GT-RXS-SKNPs produced the highest lung AUC and the longest lung MRT, indicating improved pulmonary retention. This finding suggests that the combined influence of nanoscale carrier behavior and GT coating enhanced the ability of the NPs system to maintain RXS within lung tissue while limiting systemic drug exposure. The SKNP core likely contributed to prolonged release and reduced rapid systemic availability, while the GT layer may have altered the nano–biointerface, hydration shell, protein interactions, and tissue interaction pattern after IV administration (Abdelrady et al., 2019; Thakur et al., 2023).

The therapeutic efficacy arm was consistent with the lung-distribution findings. GT-RXS-SKNPs produced the strongest improvement in histopathological injury, collagen deposition, myofibroblast activation, inflammatory cytokines, hydroxyproline content, TGF-β1, MMP-2, MMP-9, and oxidative-stress markers. These improvements were greater than those observed with free RXS or uncoated RXS-SKNPs, supporting the benefit of gelatin coating in enhancing the therapeutic performance of RXS-loaded SKNPs.

The biological response showed a consistent formulation-dependent hierarchy rather than an isolated improvement in a single endpoint. Free RXS produced partial protection, but incorporation into SKNPs strengthened the response, and GT coating further broadened the therapeutic effect. This pattern is important because pulmonary fibrosis is not represented by one biomarker alone; it requires simultaneous improvement in tissue architecture, collagen burden, myofibroblast activation, inflammatory signaling, redox balance, and matrix remodeling (Jenkins et al., 2017).

The convergence of the histological, morphometric, hydroxyproline, and α-SMA findings indicates that GT-RXS-SKNPs reduced both the structural and cellular components of fibrosis. The quantitative morphometric and biochemical endpoints were particularly valuable because semiquantitative scores such as H&E and Ashcroft can be less sensitive for detecting adjacent treatment differences. Thus, the strongest evidence for the superiority of GT-RXS-SKNPs comes from the agreement between multiple endpoint classes rather than from any single score.

The reduction in TGF-β1, α-SMA, and collagen accumulation suggests attenuation of a central profibrotic loop involving epithelial injury, fibroblast activation, myofibroblast differentiation, and extracellular matrix deposition (Flanders, 2004; Kim et al., 2018). Likewise, the decrease in total MMP-2 and MMP-9 indicates suppression of the gelatinase-associated remodeling environment induced by bleomycin (Ishida et al., 2023). However, these gelatinase data should not be overinterpreted as direct proof of *in vivo* GT-shell cleavage. They support the biological relevance of the selected MMP-enriched disease model, whereas direct confirmation of nanoparticle degradation in fibrotic lung tissue would require additional tracking or degradation-specific experiments.

The anti-inflammatory and antioxidant effects provide further mechanistic coherence. BALF cytokines and oxidative stress are closely linked to epithelial damage, fibroblast activation, and matrix deposition; therefore, simultaneous reduction of TNF-α, IL-6, IL-1β, and MDA together with restoration of GSH and SOD suggests a broader shift of the injured lung microenvironment toward resolution (Cheresh et al., 2013). This coordinated biological correction is more consistent with improved local drug delivery than with a nonspecific effect on one downstream marker.

The integrated percent-recovery analysis helped summarize this multi-endpoint response and reduced the risk of overemphasizing a single assay. The rank order of recovery paralleled the lung-distribution profile: GT-RXS-SKNPs combined the strongest pulmonary retention with the broadest biological recovery, followed by uncoated RXS-SKNPs and then free RXS. This agreement supports a delivery-driven explanation in which the SKNP core sustained RXS availability, while the GT shell added a disease-responsive interface suitable for the gelatinase-rich fibrotic microenvironment.

Nevertheless, the study should be interpreted within its mechanistic limits. Direct HIF-pathway endpoints were not included, total gelatinase abundance was measured rather than enzyme activity, and the biological evaluation was performed at a single terminal time point. Accordingly, the mechanistic conclusion should remain focused on formulation-enhanced antifibrotic efficacy, with downstream attenuation of inflammation, oxidative stress, TGF-β1-associated fibrogenesis, matrix remodeling, and myofibroblast activation. Future work incorporating time-course sampling, HIF-pathway profiling, active gelatinase assays, and *in vivo* nanoparticle tracking would further clarify the relative contribution of each mechanism.

**FIGURE 10:**
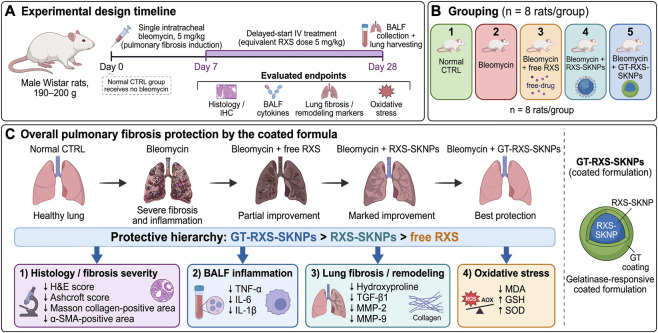
Schematic illustration of the experimental design, animal grouping, and overall pulmonary fibrosis protection achieved by GT-RXS-SKNPs in bleomycin-induced pulmonary fibrosis. **(A)** Experimental timeline showing single intratracheal bleomycin administration on day 0, delayed-start intravenous treatment from day 7 to day 28 at an equivalent RXS dose of 5 mg/kg, and terminal BALF collection and lung harvesting on day 28. The evaluated endpoint categories included histology/IHC, BALF cytokines, lung fibrosis/remodeling markers, and oxidative-stress markers. **(B)** Experimental grouping of male Wistar rats into five groups: normal control, bleomycin, bleomycin + free RXS, bleomycin + RXS-SKNPs, and bleomycin + GT-RXS-SKNPs. **(C)** Summary schematic of the overall protective hierarchy and endpoint domains assessed after treatment, including histology/fibrosis severity, BALF inflammation, lung fibrosis/remodeling markers, and oxidative stress. The schematic also illustrates the GT-RXS-SKNP coated formulation, consisting of an RXS-SKNP core surrounded by a GT coating as a gelatinase-responsive interface.

Overall, the biological evaluation complements the formulation and lung-distribution findings by showing that GT coating improved the functional therapeutic outcome of RXS-SKNPs in experimental pulmonary fibrosis. Rather than simply increasing drug exposure, the coated system produced coordinated recovery across the main pathological domains of bleomycin-induced fibrosis, supporting GT-RXS-SKNPs as a bioresponsive pulmonary delivery strategy for RXS. The overall study design together with the integrated protective hierarchy of the tested formulations is summarized in Figure 10.

## 5 Conclusion

The present study successfully developed GT-coated RXS-loaded SKNPs as a bioresponsive injectable nanoplatform for pulmonary fibrosis therapy. RXS-SKNPs were prepared by desolvation-assisted nanoprecipitation and subsequently coated with GT through electrostatic interactions. The optimized GT-RXS-SKNPs showed an increase in particle size from 145.72 ± 2.74 nm to 194.26 ± 2.94 nm and a zeta-potential shift from −30.28 ± 1.01 mV to −15.19 ± 2.38 mV, confirming surface modification while maintaining high RXS entrapment efficiency. TNBSA and FTIR analyses further supported GT coating and RXS incorporation.

The GT coating provided an MMP-2-responsive interface, as demonstrated by enzyme-triggered colloidal remodeling and enhanced RXS release under MMP-2 exposure. GT-RXS-SKNPs also showed acceptable hemocompatibility and maintained good physicochemical stability, particularly under refrigerated storage. *In vivo*, GT-RXS-SKNPs markedly improved pulmonary delivery by reducing plasma exposure, increasing lung AUC_0_–_24_ by 3.58-fold compared with free RXS and 1.71-fold compared with uncoated RXS-SKNPs, and prolonging lung MRT to 42.13 ± 8.34 h.

The therapeutic efficacy study confirmed that improved lung disposition translated into superior biological activity in bleomycin-induced pulmonary fibrosis. GT-RXS-SKNPs produced the strongest attenuation of histopathological injury, collagen deposition, myofibroblast activation, BALF inflammatory cytokines, lung hydroxyproline, TGF-β1, MMP-2, MMP-9, lipid peroxidation, and antioxidant depletion. Integrated recovery analysis further demonstrated that GT-RXS-SKNPs achieved the highest overall fibrosis recovery index, outperforming both free RXS and uncoated RXS-SKNPs. Overall, GT-RXS-SKNPs represent a promising MMP-responsive pulmonary delivery platform that enhances lung residence and therapeutic efficacy of RXS while limiting systemic exposure. These findings support further development of GT-coated SKNPs as a bioresponsive nanotherapeutic strategy for pulmonary fibrosis.

## Data Availability

The raw data supporting the conclusions of this article will be made available by the authors, without undue reservation.
