# Supplementary material for: Bioresponsive gelatin-coated silk fibroin nanoparticles enhance roxadustat therapy in experimental pulmonary fibrosis
Source: Front Pharmacol. 2026 Aug 3;17:1921860. doi: 10.3389/fphar.2026.1921860 (PMC13477923; doi:10.3389/fphar.2026.1921860)
Supplement: Supplementary file 1 [file Table1.docx]

**Rationale for SF concentration selection.**

Preliminary screening of SF concentration was performed to identify the lowest polymer concentration capable of producing homogeneous RXS-SKNPs with suitable nanoscale size, acceptable PDI, stable surface charge, and adequate drug entrapment (**Table S1)**. Although 0.125% w/v SF produced the smallest particles, the EE% was markedly low, indicating insufficient polymeric matrix capacity for efficient RXS incorporation. Increasing SF concentration to 0.25% w/v markedly improved EE% while maintaining a small particle size, acceptable PDI, and a highly negative zeta potential, indicating good colloidal stability. Further increasing SF concentration to 0.5% w/v produced only a limited additional increase in EE%, but caused a pronounced increase in particle size and PDI, suggesting the formation of larger and more heterogeneous nanoparticles. At 1% w/v SF, phase separation occurred, indicating poor formulation stability under the selected preparation conditions. Therefore, 0.25% w/v SF was selected as the optimal concentration because it provided the best balance between nanoscale particle size, colloidal homogeneity, surface stability, and RXS entrapment efficiency.

**Table S1**. Preliminary screening of silk fibroin concentration for preparation of RXS-SKNPs.

| SF concentration | Appearance | PS (nm) | PDI | ZP (mV) | EE (%) |
| --- | --- | --- | --- | --- | --- |
| 0.125 % w/v | Homogenous | 107.35 ± 3.66 | 0.218 ± 0.005 | -24.83 ± 0.98 | 49.33 ± 2.06 |
| 0.25 % w/v | Homogenous | 145.72 ± 2.74 | 0.253 ± 0.031 | -30.28 ± 1.01 | 73.59 ± 1.25 |
| 0.5 % w/v | Homogenous | 406.17 ± 5.11 | 0.426 ± 0.029 | -33.15 ± 1.27 | 76.19 ± 0.97 |
| 1 % w/v | Phase separation | - | - | - | - |
